# Supplementary material for: Positive interactions within and between populations decrease the likelihood of evolutionary rescue
Source: PLoS Comput Biol. 2021 Feb 18;17(2):e1008732. doi: 10.1371/journal.pcbi.1008732 (PMC7924792; doi:10.1371/journal.pcbi.1008732)
Supplement: S1 Text — Section A: Modeling Assumptions. Section B: Models. Section C: Theoretical analysis. Section D: Models with continuous Allee effect. Section E: Positive interactions provide a fitness advantage. Section F: Model of mutualism in which populations are affected at high densities. Table A: Parameters table. (DOCX) [file pcbi.1008732.s001.docx]

**Supplementary information for**

**Positive interactions within and between populations decrease the likelihood of evolutionary rescue**

Yaron Goldberg & Jonathan Friedman

Department of Plant Pathology and Microbiology, The Hebrew University of Jerusalem, Rehovot, Israel.

[**Section A: Modeling Assumptions**](#_heading=h.3znysh7) **[2](#_heading=h.3znysh7)**

[**Section B: Models**](#_heading=h.tyjcwt) **[3](#_heading=h.tyjcwt)**

[Intraspecies cooperation](#_heading=h.3dy6vkm) [3](#_heading=h.3dy6vkm)

[Mutualism](#_heading=h.1t3h5sf) [4](#_heading=h.1t3h5sf)

[Cheaters](#_heading=h.4d34og8) [5](#_heading=h.4d34og8)

[**Section C: Theoretical analysis**](#_heading=h.17dp8vu) **[6](#_heading=h.17dp8vu)**

[Intraspecies cooperation](#_heading=h.3rdcrjn) [6](#_heading=h.3rdcrjn)

[Mutualism](#_heading=h.26in1rg) [8](#_heading=h.26in1rg)

[**Section D: Models with continuous Allee effect**](#_heading=h.35nkun2) **[10](#_heading=h.35nkun2)**

[Intraspecies cooperation](#_heading=h.1ksv4uv) [10](#_heading=h.1ksv4uv)

[Mutualism](#_heading=h.44sinio) [11](#_heading=h.44sinio)

[Cheaters - non-oscillatory model](#_heading=h.z337ya) [12](#_heading=h.z337ya)

[**Section E: Positive interactions provide a fitness advantage**](#_heading=h.1y810tw) **[14](#_heading=h.1y810tw)**

[Comparison of non cooperating populations and interspecies cooperation](#_heading=h.4i7ojhp) [14](#_heading=h.4i7ojhp)

[Interspecies cooperation and mutualism](#_heading=h.2xcytpi) [15](#_heading=h.2xcytpi)

[**Section F: Model of mutualism in which populations are affected at high densities**](#_heading=h.3whwml4) **[17](#_heading=h.3whwml4)**

[**Table**](#_heading=h.2bn6wsx) **A 18**

#

# Section A: Modeling Assumptions

To enable analytical calculations and maintain simplicity, we have constructed a minimal model that does not consider many aspects that may lead to more rich and complex adaptive dynamics. First, our model includes only a single type of mutations that improve the growth rate by a fixed amount. We have not considered variations in the effect of mutations on the growth rate, mutations that impact the positive interactions themselves (e.g. by changing the critical population size), or the possibility of epistatic mutations. Moreover, our model does not include stochastic effects due to finite population size, which are expected to lead to the extinction of some of the adapted mutants due to drift. In addition, the spatial structure of populations, which is known to have an important role in shaping the evolutionary dynamics within and between species. Finally, we’ve considered linear interactions and a step function Allee effect, whereas natural interactions may have more complex functional forms. Nonetheless, we believe that our qualitative results are robust to these assumptions and hold true also in more complex models. Such robustness is exemplified by our analysis of a continuous Allee effect, where similar qualitative results occur even when positive interactions are modeled using a more complex function than the simple one used in the main text (section 4 in the Supplementary Information).

# Section B: Models

## Intraspecies cooperation

The model is based on the classical logistic growth model, in which populations initially grow at rate [
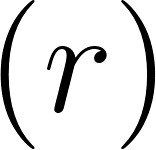
](https://www.codecogs.com/eqnedit.php?latex=(r)#0) and saturate at carrying capacity [
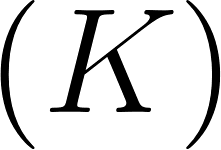
](https://www.codecogs.com/eqnedit.php?latex=(K)#0) . It extends the logistic model by applying a strong Allee effect through the reduction of individuals’ growth rate when the population is below a critical size [
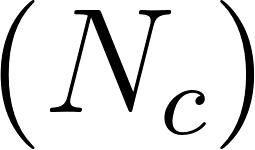
](https://www.codecogs.com/eqnedit.php?latex=(N_c)#0) . In addition, we included a death rate [
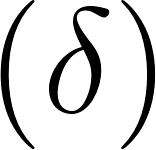
](https://www.codecogs.com/eqnedit.php?latex=(%5Cdelta)#0) reflecting an external environmental stress that is independent of the interactions within the populations. The model includes an ancestral population and a mutant population with an increased growth rate . The dynamics of the ancestor and mutant populations are given by (**Fig. S1**):

[
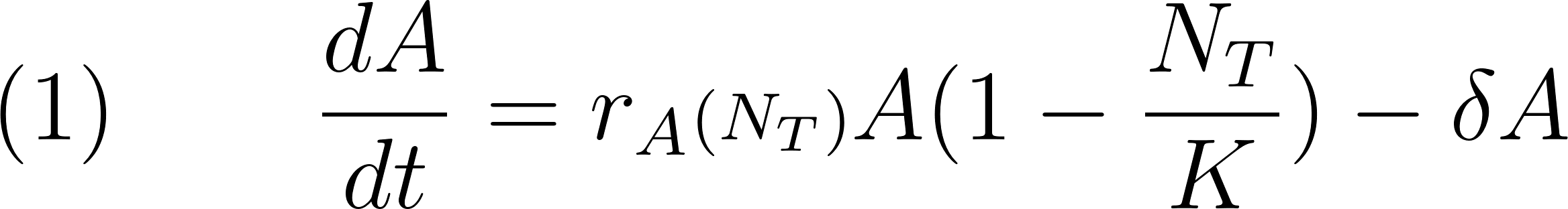
](https://www.codecogs.com/eqnedit.php?latex=(1)%20%5Cquad%20%5Cquad%20%20%5Cfrac%7BdA%7D%7Bdt%7D%20%3D%20r_%7BA%7D%7B%5Cscriptstyle%20(N_T)%7D%20A%20%20(1-%5Cfrac%7BN_T%7D%7BK%7D)%20-%20%5Cdelta%20A#0)

[
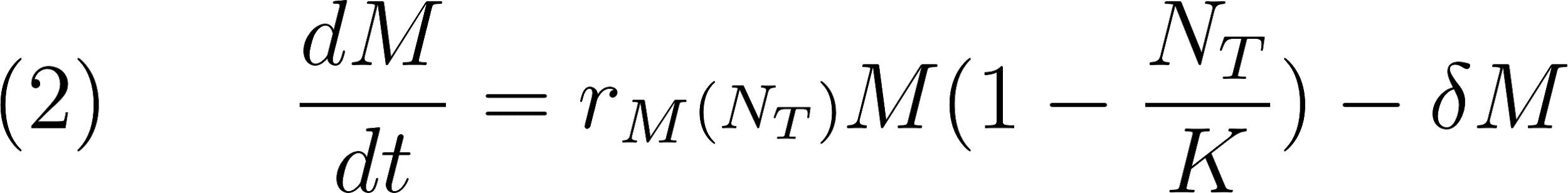
](https://www.codecogs.com/eqnedit.php?latex=(2)%20%5Cquad%20%5Cquad%20%20%5Cfrac%7BdM%7D%7Bdt%7D%20%3D%20r_%7BM%7D%7B%5Cscriptstyle%20(N_T)%7D%20M%20%20(1-%5Cfrac%7BN_T%7D%7BK%7D)%20-%20%5Cdelta%20M#0).

The growth rate [
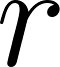
](https://www.codecogs.com/eqnedit.php?latex=r#0) depends on the total population size [
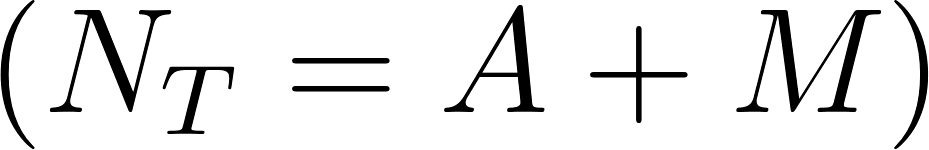
](https://www.codecogs.com/eqnedit.php?latex=(N_T%3DA%2BM)#0):

[
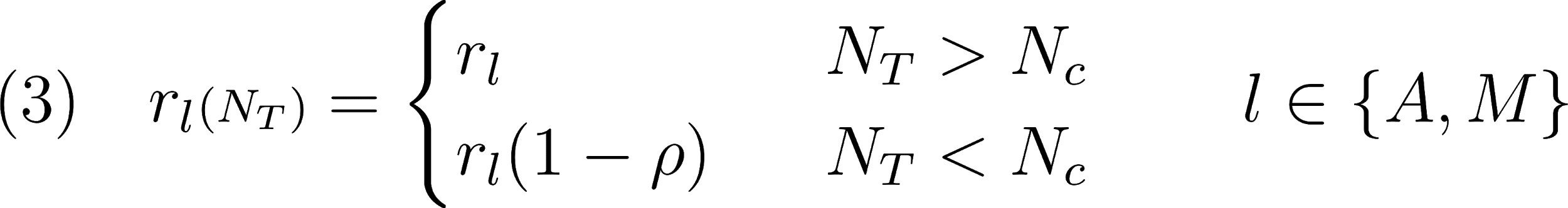
](https://www.codecogs.com/eqnedit.php?latex=(3)%20%5Cquad%20r_%7Bl%7D%7B%5Cscriptstyle%20(N_T)%7D%3D%20%5Cbegin%7Bcases%7D%20r_l%20%26%20%5C%20%5C%20%20N_T%3EN_c%20%5C%5Cr_l(1-%5Crho%20)%20%26%20%5C%20%5C%20N_T%3CN_c%20%5Cend%7Bcases%7D%20%5Cquad%20%5Cquad%20%7B%5Cscriptsize%20l%20%5Cin%20%5C%7BA%2CM%5C%7D%7D#0),

where [
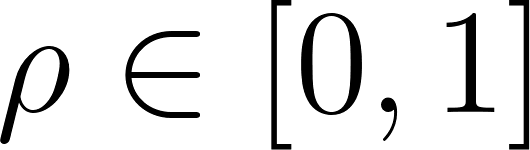
](https://www.codecogs.com/eqnedit.php?latex=%5Crho%20%5Cin%20%5B0%2C1%5D#0) is the fraction by which growth rates decrease below the critical population size [
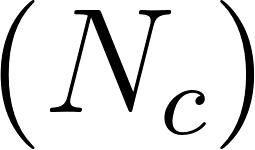
](https://www.codecogs.com/eqnedit.php?latex=(N_c)#0).


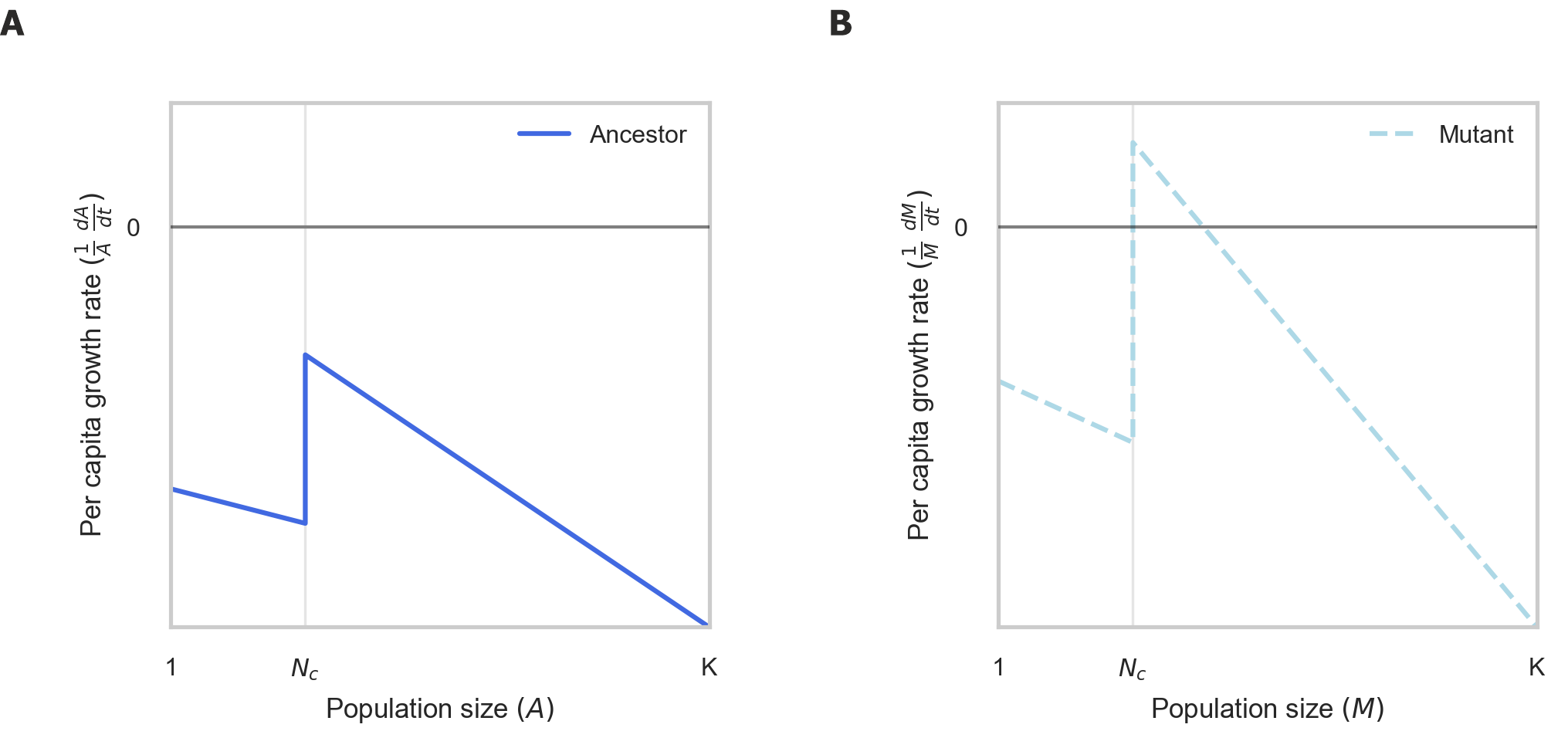


**Figure S1: Change in per capita growth rate of intraspecies cooperating populations.** (A) Ancestor per capita growth rate as a function of total population size after stress onset. Growth rate decreases with population size due to intraspecies competition. When the population size is below the critical population size [
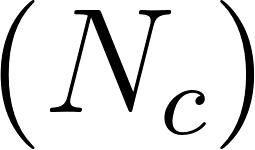
](https://www.codecogs.com/eqnedit.php?latex=(N_c)#0), the growth rate reduces further due to the Allee effect. Ancestor growth rate is always negative due to environmental stress. (B) Mutant per capita growth rate as a function of total population size after stress onset. Here, growth rate can be positive above [
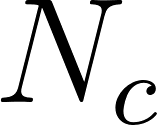
](https://www.codecogs.com/eqnedit.php?latex=N_c#0) due to higher growth rate [
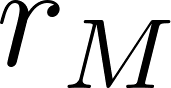
](https://www.codecogs.com/eqnedit.php?latex=r_M#0). Thus, survival is possible when the total population size is sufficiently high.

Each simulation begins with the growth of an ancestor population starting at carrying capacity [
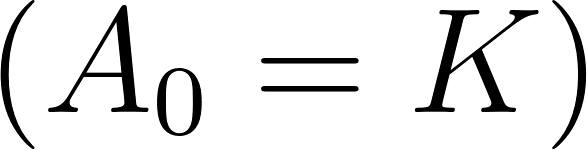
](https://www.codecogs.com/eqnedit.php?latex=(A_0%3DK)#0) in an unstressed environment [
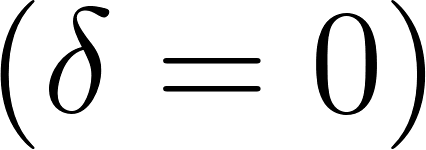
](https://www.codecogs.com/eqnedit.php?latex=(%5Cdelta%20%3D%200)#0), and without any mutants [
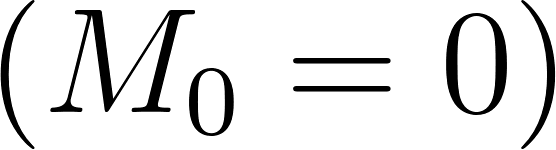
](https://www.codecogs.com/eqnedit.php?latex=(M_0%3D0)#0). At stress onset, the death rate is increased such that it exceeds the ancestral exponential growth rate ([
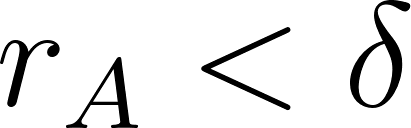
](https://www.codecogs.com/eqnedit.php?latex=r_A%3C%5Cdelta#0)), leading the population to decline toward extinction. Mutation events are modeled as a Poisson process, with the expected number of mutation events occurring during a time interval [
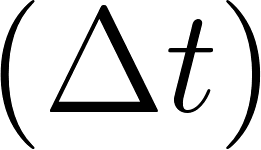
](https://www.codecogs.com/eqnedit.php?latex=(%5CDelta%20t)#0) given by the ancestral population size and mutation rate ($\mu$):

[
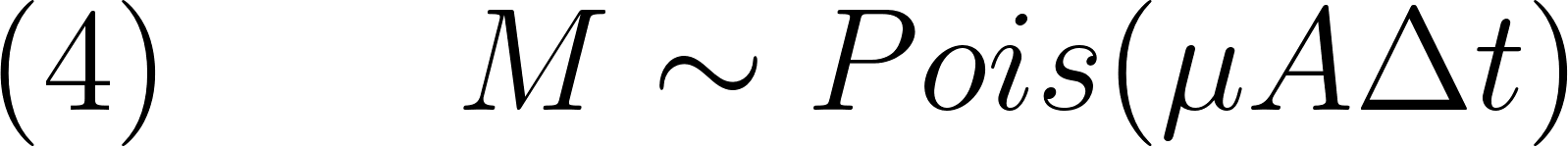
](https://www.codecogs.com/eqnedit.php?latex=(4)%20%5Cquad%20%5Cquad%20M%20%5Csim%20Pois(%5Cmu%20A%20%5CDelta%20t)#0)

Mutants differ from ancestors only in their elevated exponential growth rate [
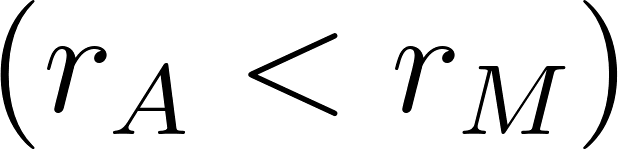
](https://www.codecogs.com/eqnedit.php?latex=(r_A%3Cr_M)#0), which allows them to survive the stress, but only when the total population size exceeds the critical threshold [
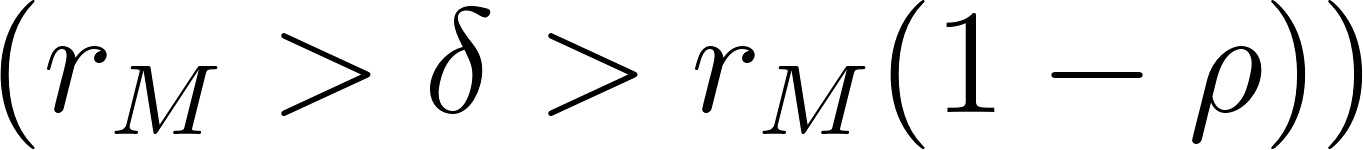
](https://www.codecogs.com/eqnedit.php?latex=(r_M%3E%20%5Cdelta%20%3E%20r_M(1%20-%20%5Crho))#0). For simplicity, no further stochastic effects were considered in this model.

We assessed the evolutionary rescue probability by calculating the fraction of simulations in which the mutants were able to spread and exceed the critical population size:

[
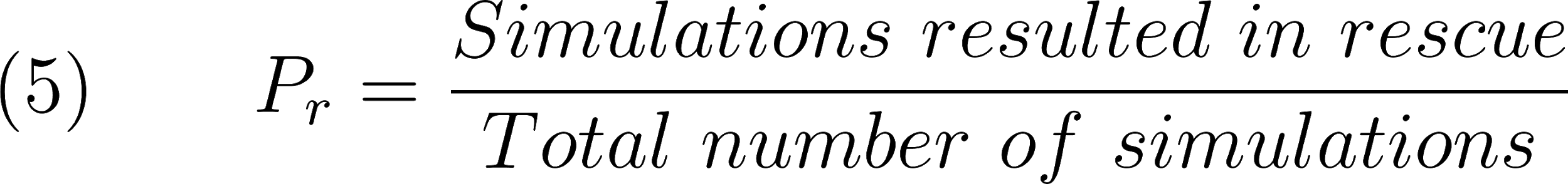
](https://www.codecogs.com/eqnedit.php?latex=(5)%20%5Cquad%20%5Cquad%20P_r%20%3D%20%5Cfrac%7BSimulations%20%5C%20resulted%20%5C%20in%20%5C%20rescue%7D%7BTotal%20%5C%20number%20%5C%20of%20%5C%20simulations%7D#0)

Here, we run 1000 simulations for each parameters combination (**Table A**). Simulations were stopped when an extinction [
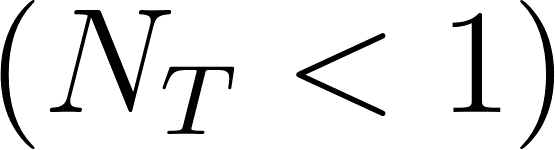
](https://www.codecogs.com/eqnedit.php?latex=(N_T%3C1)#0) or rescue [
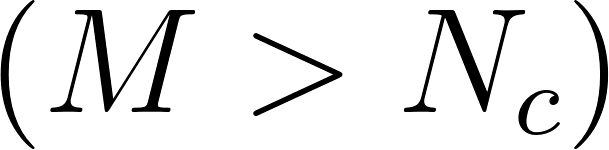
](https://www.codecogs.com/eqnedit.php?latex=(M%3EN_c)#0) were observed.

## Mutualism

We have used an extended model in which two species are dependent on each other in an obligatory manner. Analogously to the case of intraspecies cooperation, the growth rate of each species is reduced when the population size of its partner is below a critical population size ($N_{c}$):

[
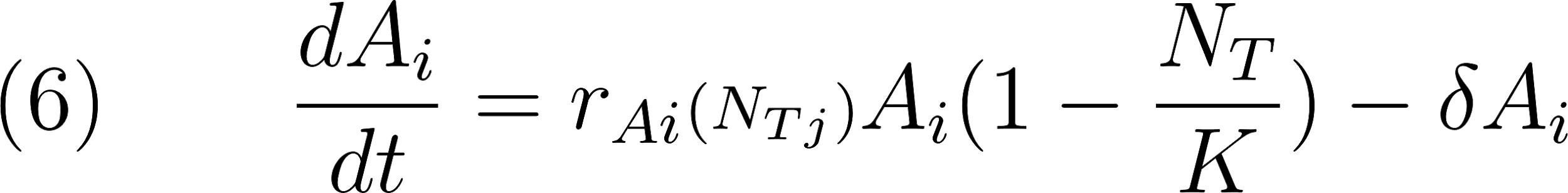
](https://www.codecogs.com/eqnedit.php?latex=(6)%20%5Cquad%20%5Cquad%20%20%5Cfrac%7BdA_i%7D%7Bdt%7D%20%3D%20r_%7BAi%7D%7B%5Cscriptstyle%20(N_%7BTj%7D)%20%7D%20%20A_i%20%20(1-%5Cfrac%7BN_T%7D%7BK%7D)%20-%20%5Cdelta%20A_i#0)

[
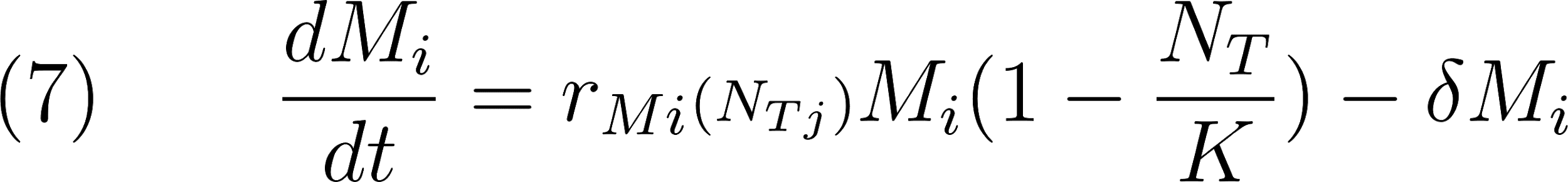
](https://www.codecogs.com/eqnedit.php?latex=(7)%20%5Cquad%20%5Cquad%20%20%5Cfrac%7BdM_i%7D%7Bdt%7D%20%3D%20r_%7BMi%7D%7B%5Cscriptstyle%20(N_T_j)%20%7D%20%20M_i%20%20(1-%5Cfrac%7BN_T%7D%7BK%7D)%20-%20%5Cdelta%20M_i#0)

[
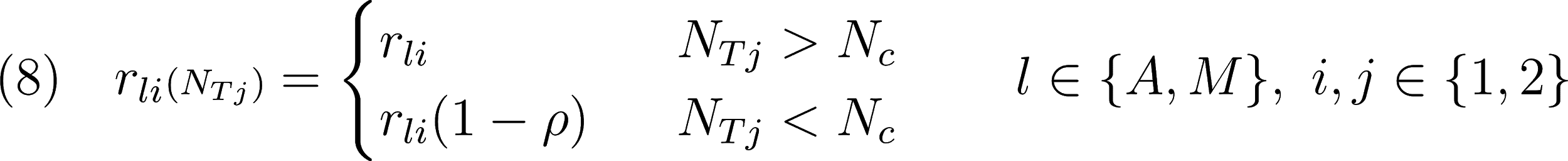
](https://www.codecogs.com/eqnedit.php?latex=(8)%20%5Cquad%20r_%7Bli%7D%7B%5Cscriptstyle%20(N_%7BTj%7D)%7D%3D%20%5Cbegin%7Bcases%7D%20r_%7Bli%7D%20%26%20%5C%20%5C%20%20N_%7BTj%7D%3EN_c%20%5C%5Cr_%7Bli%7D(1-%5Crho%20)%20%26%20%5C%20%5C%20N_%7BTj%7D%3CN_c%20%5Cend%7Bcases%7D%20%5Cquad%20%5Cquad%20%7B%5Cscriptsize%20l%20%5Cin%20%5C%7BA%2CM%5C%7D%7D%2C%5C%20%7B%5Cscriptsize%20i%2Cj%20%5Cin%20%5C%7B1%2C2%5C%7D%7D#0)

Where [
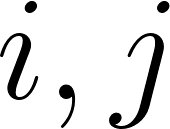
](https://www.codecogs.com/eqnedit.php?latex=i%2Cj#0) represents the two different species. The simulation process is similar to the one described for intraspecies cooperation, where each species’ initial population density is [
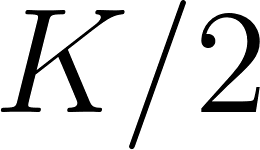
](https://www.codecogs.com/eqnedit.php?latex=K%2F2#0) and we consider [
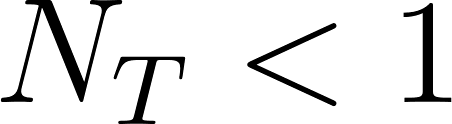
](https://www.codecogs.com/eqnedit.php?latex=N_T%3C1#0) as extinction, and [
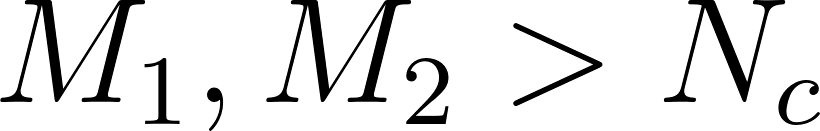
](https://www.codecogs.com/eqnedit.php?latex=M_1%2CM_2%3EN_c#0) as rescue.

In order to explore the effect of mutualism with no competitive effect, we altered equations 6,7 by replacing [
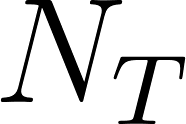
](https://www.codecogs.com/eqnedit.php?latex=N_T#0), the total population size of all species, with [
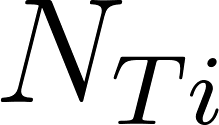
](https://www.codecogs.com/eqnedit.php?latex=N_%7BTi%7D#0), the population size of species [
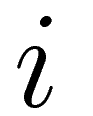
](http://www.sciweavers.org/tex2img.php?bc=Transparent&fc=Black&im=jpg&fs=100&ff=modern&edit=0&eq=i#0).

## Cheaters

We adapted a previously established model that describes the dynamics of cooperators and cheaters^1^. In this model, both cooperators and cheaters are affected by the cooperator’s population density, and have a reduced growth rate when the cooperator population is below a critical size. However, cooperators and cheaters coexist since cheaters have a growth advantage ([
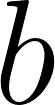
](https://www.codecogs.com/eqnedit.php?latex=b#0)) at high cooperator density since they do not pay the cost of cooperation, whereas at low populations densities cooperators have a growth advantage ([
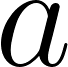
](https://www.codecogs.com/eqnedit.php?latex=a#0)) at low populations densities, reflecting their preferential access to the public goods they produce:

[
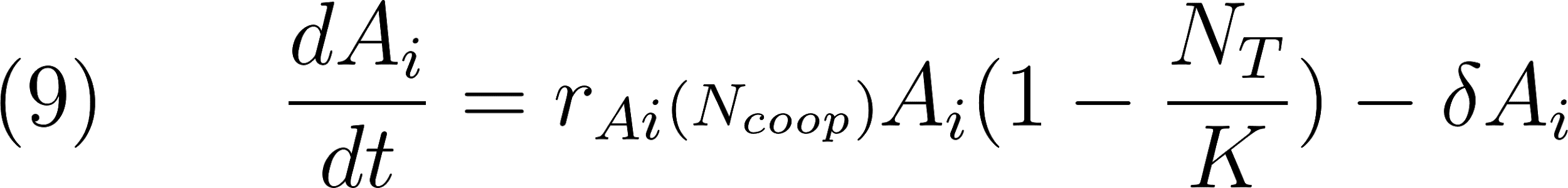
](https://www.codecogs.com/eqnedit.php?latex=(9)%20%5Cquad%20%5Cquad%20%20%5Cfrac%7BdA_i%7D%7Bdt%7D%20%3D%20r_%7BAi%7D%7B%5Cscriptstyle%20(N_%7Bcoop%7D)%20%7D%20%20A_i%20%20(1-%5Cfrac%7BN_T%7D%7BK%7D)%20-%20%5Cdelta%20A_i#0)

[
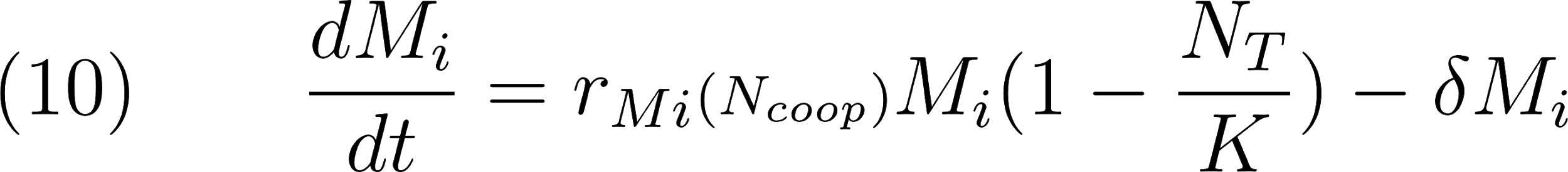
](https://www.codecogs.com/eqnedit.php?latex=(10)%20%5Cquad%20%5Cquad%20%20%5Cfrac%7BdM_i%7D%7Bdt%7D%20%3D%20r_%7BMi%7D%7B%5Cscriptstyle%20(N_%7Bcoop%7D)%20%7D%20%20M_i%20%20(1-%5Cfrac%7BN_T%7D%7BK%7D)%20-%20%5Cdelta%20M_i#0)

[
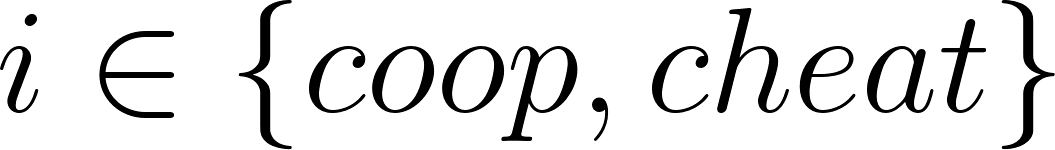
](https://www.codecogs.com/eqnedit.php?latex=%7B%5Cscriptsize%20i%20%5Cin%20%5C%7Bcoop%2Ccheat%5C%7D%7D#0)

[
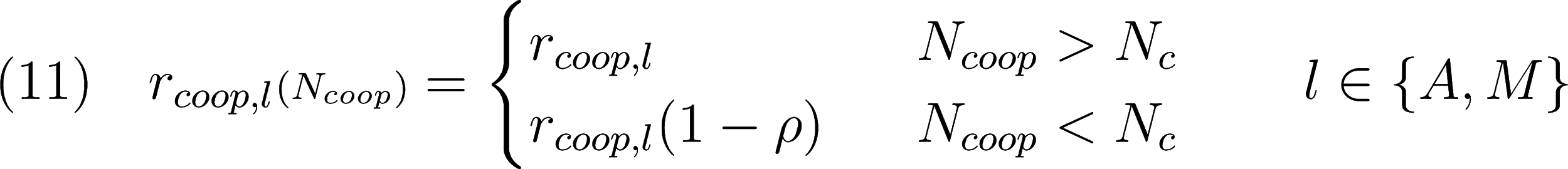
](https://www.codecogs.com/eqnedit.php?latex=(11)%20%5Cquad%20r_%7Bcoop%2Cl%7D%7B%5Cscriptstyle%20(N_%7Bcoop%7D)%7D%3D%20%5Cbegin%7Bcases%7D%20r_%7Bcoop%2Cl%7D%20%26%20%5C%20%5C%20%20N_%7Bcoop%7D%3EN_c%20%5C%5Cr_%7Bcoop%2Cl%7D(1-%5Crho%20)%20%26%20%5C%20%5C%20N_%7Bcoop%7D%3CN_c%20%5Cend%7Bcases%7D%20%5Cquad%20%5Cquad%20%7B%5Cscriptsize%20l%20%5Cin%20%5C%7BA%2CM%5C%7D%7D#0)

[
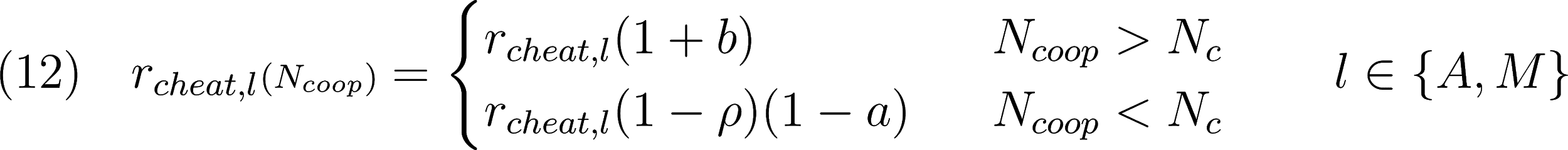
](https://www.codecogs.com/eqnedit.php?latex=(12)%20%5Cquad%20r_%7Bcheat%2Cl%7D%7B%5Cscriptstyle%20(N_%7Bcoop%7D)%7D%3D%20%5Cbegin%7Bcases%7D%20r_%7Bcheat%2Cl%7D(1%2Bb)%20%26%20%5C%20%5C%20%20N_%7Bcoop%7D%3EN_c%20%5C%5Cr_%7Bcheat%2Cl%7D(1-%5Crho%20)(1-a)%20%26%20%5C%20%5C%20N_%7Bcoop%7D%3CN_c%20%5Cend%7Bcases%7D%20%5Cquad%20%5Cquad%20%7B%5Cscriptsize%20l%20%5Cin%20%5C%7BA%2CM%5C%7D%7D#0)

Where [
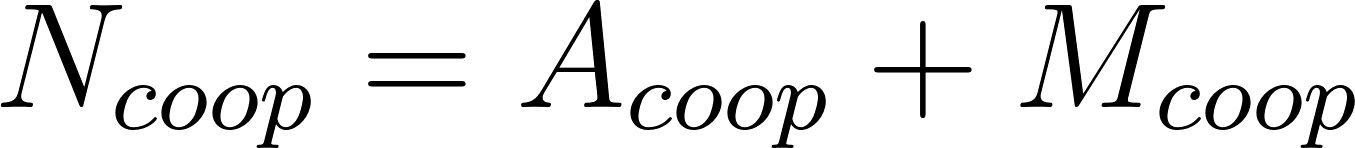
](https://www.codecogs.com/eqnedit.php?latex=N_%7Bcoop%7D%3DA_%7Bcoop%7D%2BM_%7Bcoop%7D#0) is the total population size of the cooperators.

Here, simulations begin with initial population size [
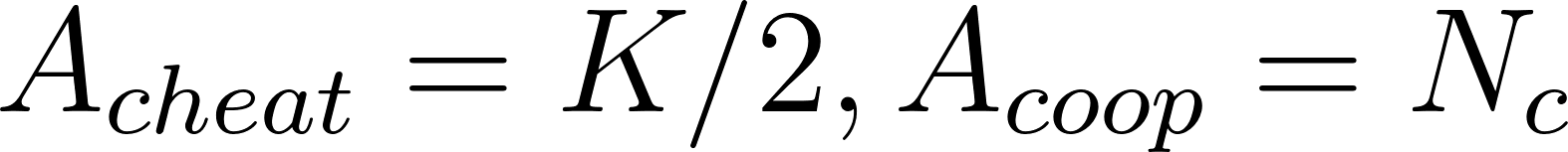
](https://www.codecogs.com/eqnedit.php?latex=A_%7Bcheat%7D%3DK%2F2%2C%20A_%7Bcoop%7D%3DN_c#0) and low external death rate [
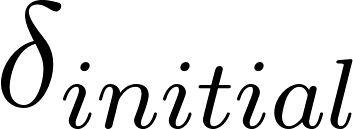
](https://www.codecogs.com/eqnedit.php?latex=%5Cdelta_%7Binitial%7D#0) that allows oscillatory dynamics. At stress onset, external death rate changes to [
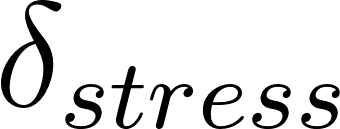
](https://www.codecogs.com/eqnedit.php?latex=%5Cdelta_%7Bstress%7D#0) and mutation events occur as in the intraspecies model. Since the dynamics prior to stress onset are oscillatory, stress onset was sampled uniformly over a single period of the oscillation after the dynamics stabilized. Rescue probability was calculated as in the intraspecies model, and we considered [
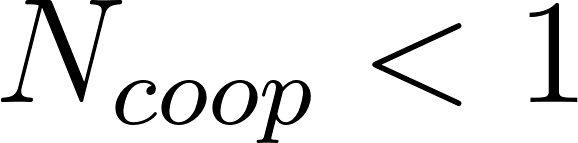
](https://www.codecogs.com/eqnedit.php?latex=N_%7Bcoop%7D%3C1#0) as extinction, and [
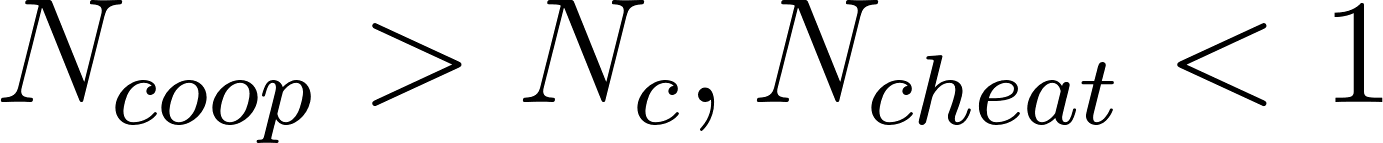
](https://www.codecogs.com/eqnedit.php?latex=N_%7Bcoop%7D%3EN_c%2CN_%7Bcheat%7D%3C1#0) as rescue.

##

# Section C: Theoretical analysis

## Intraspecies cooperation

We have formulated a theoretical analysis of evolutionary rescue probability in populations engaged with intraspecies cooperation. Since rescue probability is dependent on the rescue time window ([
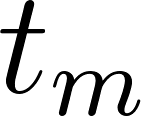
](https://www.codecogs.com/eqnedit.php?latex=t_m#0)) - the time window during which adapted mutants can rise and prevent the population’s extinction - we constructed an approximation of this time window first. Here we approximate [
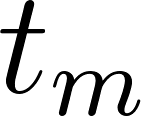
](https://www.codecogs.com/eqnedit.php?latex=t_m#0) by calculating the difference between the time it takes the ancestral population to decline to the critical population size, and the time it takes adapted mutants to grow sufficiently in order to rescue the population from collapse. We define [
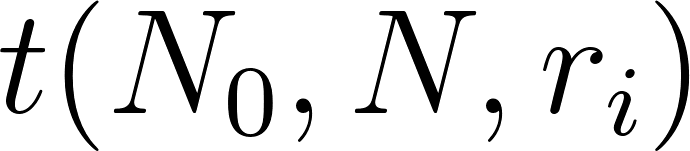
](https://www.codecogs.com/eqnedit.php?latex=t(N_0%2CN%2Cr_i)%250) as the time it takes a species with growth rate [
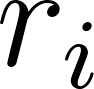
](https://www.codecogs.com/eqnedit.php?latex=r_i%250) to get from initial density [
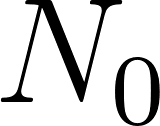
](https://www.codecogs.com/eqnedit.php?latex=N_0%250) to final density [
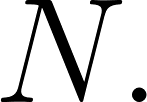
](https://www.codecogs.com/eqnedit.php?latex=N.#0) Thus, in order to calculate [
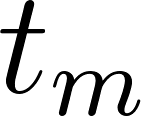
](https://www.codecogs.com/eqnedit.php?latex=t_m#0) we need:

[
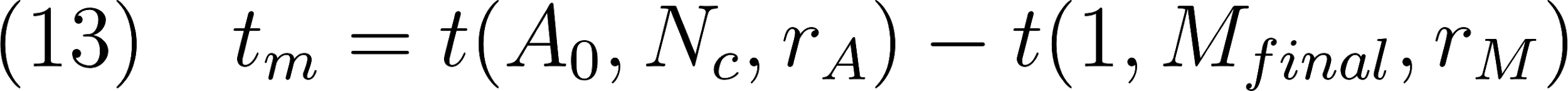
](https://www.codecogs.com/eqnedit.php?latex=(13)%20%5Cquad%20t_m%20%3D%20t(A_%7B0%7D%2CN_%7Bc%7D%2Cr_A)%20-%20t(1%2CM_%7Bfinal%7D%2Cr_%7BM%7D)%20#0)

Where [
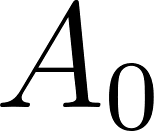
](https://www.codecogs.com/eqnedit.php?latex=A_0#0) is the initial population size of the ancestor population, [
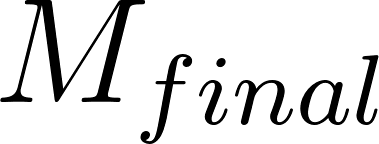
](https://www.codecogs.com/eqnedit.php?latex=M_%7Bfinal%7D#0) is the mutant population size that is sufficient in order to rescue the population, and [
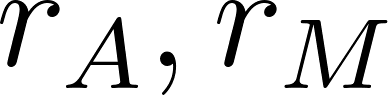
](https://www.codecogs.com/eqnedit.php?latex=r_A%2Cr_M#0) are the ancestor and mutant growth rates.

We begin with approximating the time it takes the ancestral population to decline to the critical population size [
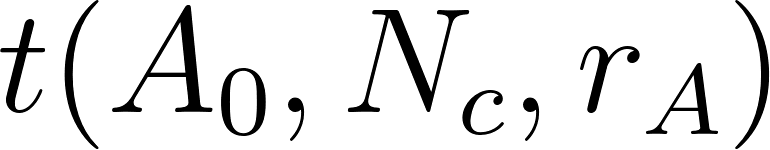
](https://www.codecogs.com/eqnedit.php?latex=t(A_%7B0%7D%2CN_%7Bc%7D%2Cr_A)#0). In order to find [
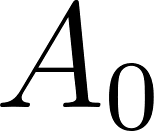
](https://www.codecogs.com/eqnedit.php?latex=A_0#0), we note that while the ancestor population density prior to stress onset is [
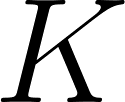
](https://www.codecogs.com/eqnedit.php?latex=K#0), mutations can grow only when the ancestor population density decreases. Hence, we set [
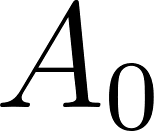
](https://www.codecogs.com/eqnedit.php?latex=A_0#0) to be the maximal ancestor population density for which a single mutant has a non-negative growth rate (eq. 2):

[
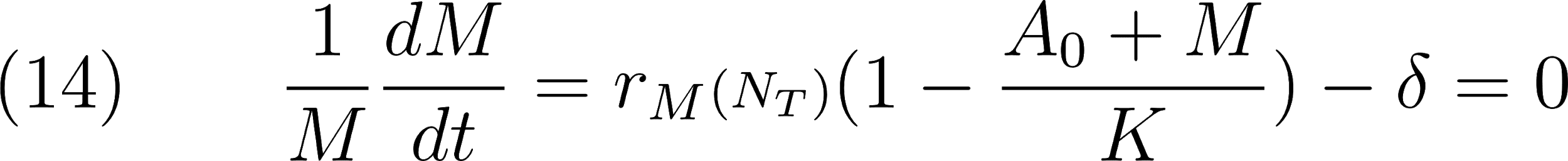
](https://www.codecogs.com/eqnedit.php?latex=(14)%20%5Cquad%20%5Cquad%20%20%5Cfrac%7B1%7D%7BM%7D%5Cfrac%7BdM%7D%7Bdt%7D%20%3D%20r_%7BM%7D%7B%5Cscriptstyle%20(N_T)%7D(1-%5Cfrac%7BA_0%2BM%7D%7BK%7D)%20-%20%5Cdelta%20%3D%200#0)

We note that since mutants can not grow up to this point, we can set [
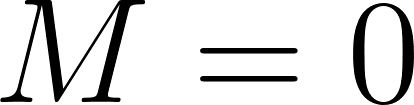
](https://www.codecogs.com/eqnedit.php?latex=M%3D0#0). In addition,[
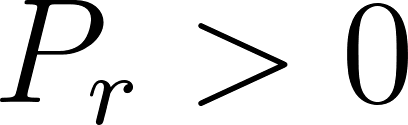
](https://www.codecogs.com/eqnedit.php?latex=P_r%3E0#0) implies [
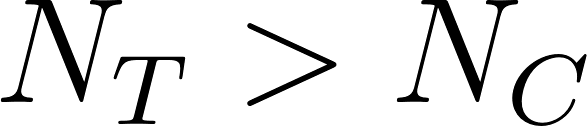
](https://www.codecogs.com/eqnedit.php?latex=N_T%3EN_C#0). Thus the solution to the equation is:

[
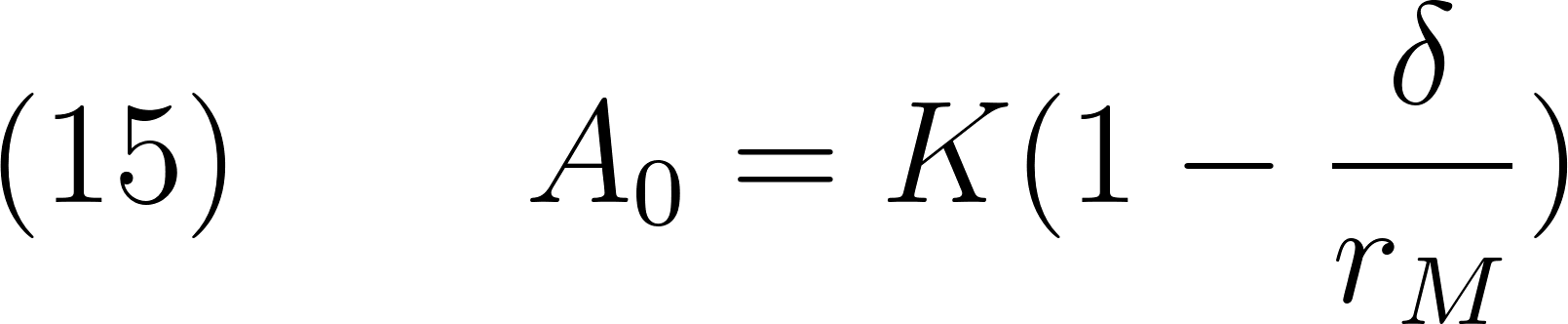
](https://www.codecogs.com/eqnedit.php?latex=(15)%20%5Cquad%20%5Cquad%20A_0%20%3D%20K(1-%5Cfrac%7B%5Cdelta%7D%7Br_M%7D)#0)

Next, we calculate the time term in our intraspecies model (eq. 1) by neglecting competition between ancestor and mutant. In addition, we note that [
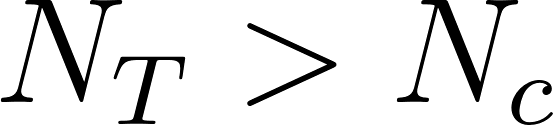
](https://www.codecogs.com/eqnedit.php?latex=N_T%3EN_c#0) during the time interval of interest. Thus, we can approximate the ancestor’s dynamics as simple logistic growth:

[
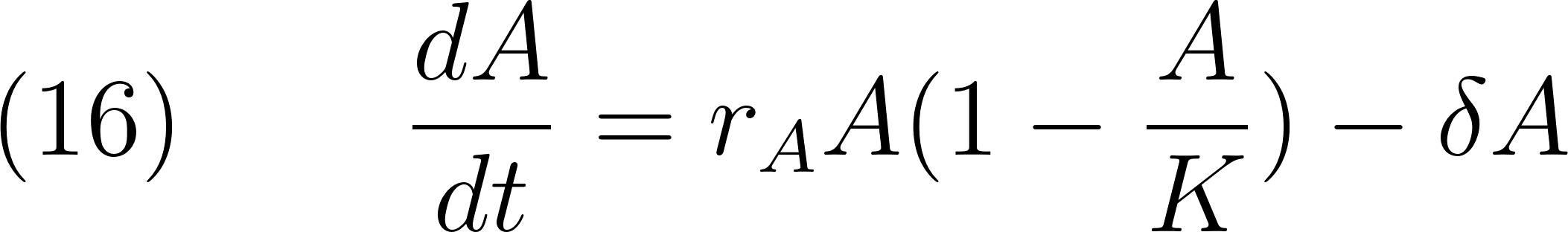
](https://www.codecogs.com/eqnedit.php?latex=(16)%20%5Cquad%20%5Cquad%20%20%5Cfrac%7BdA%7D%7Bdt%7D%20%3D%20r_%7BA%7D%20A%20%20(1-%5Cfrac%7BA%7D%7BK%7D)%20-%20%5Cdelta%20A#0)

By setting [
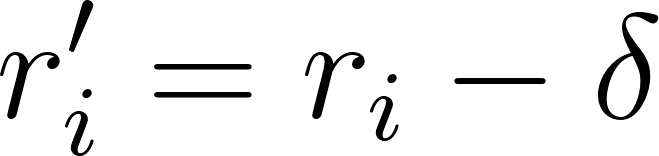
](https://www.codecogs.com/eqnedit.php?latex=r_i'%20%3D%20r_i-%20%5Cdelta#0) and [
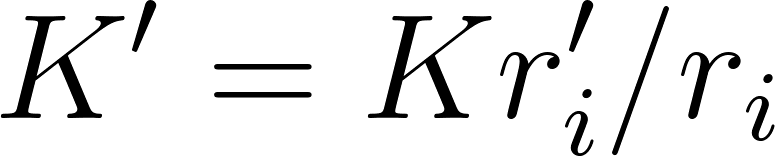
](https://www.codecogs.com/eqnedit.php?latex=K'%20%3D%20Kr_i'%2Fr_i#0) , the time term can be calculated:

[
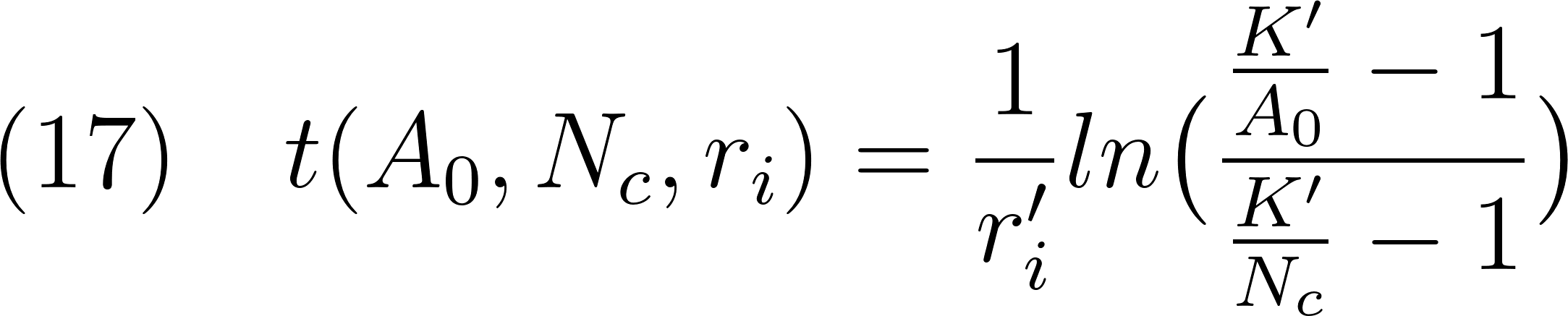
](https://www.codecogs.com/eqnedit.php?latex=(17)%20%5Cquad%20%20t(A_0%2CN_c%2Cr_i)%20%3D%20%5Cfrac%7B1%7D%7Br_i'%7Dln%5Cbig(%5Cfrac%7B%5Cfrac%7BK'%7D%7BA_0%7D-1%7D%7B%5Cfrac%7BK'%7D%7BN_c%7D-1%7D%5Cbig)#0)

Next, we want to calculate [
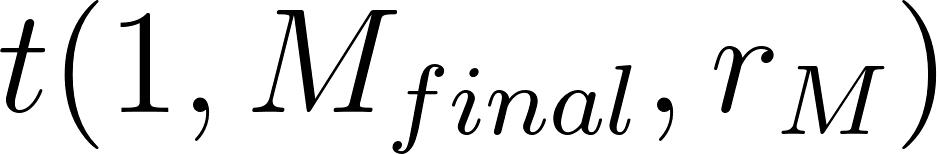
](https://www.codecogs.com/eqnedit.php?latex=t(1%2CM_%7Bfinal%7D%2Cr_%7BM%7D)#0), that is the time it takes adapted mutants to grow to a sufficient size for rescuing the population from collapse. We note that to prevent collapse the total population size must not fall below the critical population size. A sufficient condition is that the total population size increases when the ancestral population reaches the critical population size. The minimal value of [
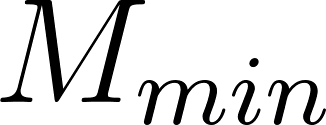
](https://www.codecogs.com/eqnedit.php?latex=M_%7Bmin%7D#0) which fulfils this condition is given by:

[
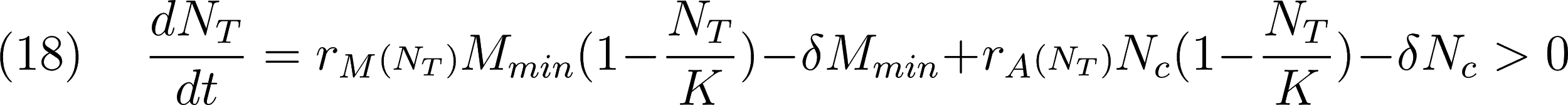
](https://www.codecogs.com/eqnedit.php?latex=(18)%20%5C%20%5Cquad%20%20%5Cfrac%7BdN_T%7D%7Bdt%7D%20%3D%20r_%7BM%7D%7B%5Cscriptstyle%20(N_T)%7D%20M_%7Bmin%7D%20%20(1-%5Cfrac%7BN_T%7D%7BK%7D)%20-%20%5Cdelta%20M_%7Bmin%7D%20%2B%20%20r_%7BA%7D%7B%5Cscriptstyle%20(N_T)%7D%20N_c%20(1-%5Cfrac%7BN_T%7D%7BK%7D)%20-%20%5Cdelta%20N_c%20%3E%200#0)

This equation does not have a closed-form solution, however it can be calculated numerically. It is also sufficient that the mutant reaches the critical population size before the ancestor does. Therefore, we chose [
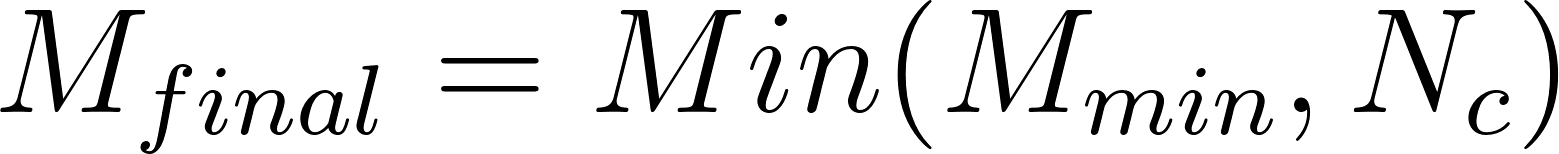
](https://www.codecogs.com/eqnedit.php?latex=M_%7Bfinal%7D%3DMin(M_%7Bmin%7D%2CN_c)#0) as the final population size.

Since the mutant population is small during the rescue time window, we neglect the competitive effect of the mutant on the ancestral population. However, the ancestor's population size is large and its competitive effect on the mutant can not be neglected. Therefore, the time for the mutant to reach [
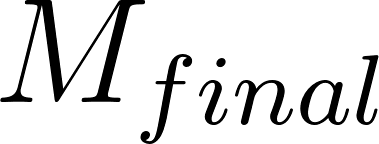
](https://www.codecogs.com/eqnedit.php?latex=M_%7Bfinal%7D#0) has to be extracted numerically from the equation:

[
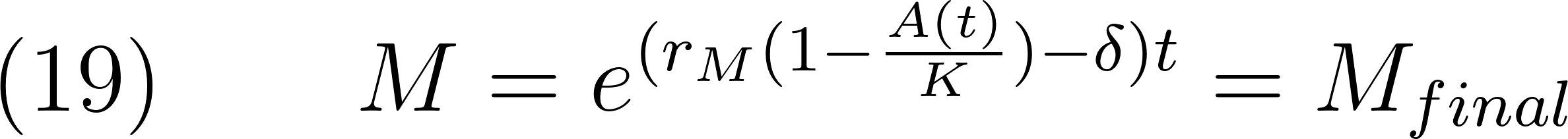
](https://www.codecogs.com/eqnedit.php?latex=(19)%20%5Cquad%20%5Cquad%20M%20%3D%20e%5E%7B(r_M(1-%5Cfrac%7BA(t)%7D%7BK%7D)-%5Cdelta)t%7D%20%3D%20M_%7Bfinal%7D#0)

After calculating the rescue time window, the expected number of mutations that occur within this time window can be calculated by integrating over the ancestor population density during the rescue time window and multiplying by the mutation rate:

[
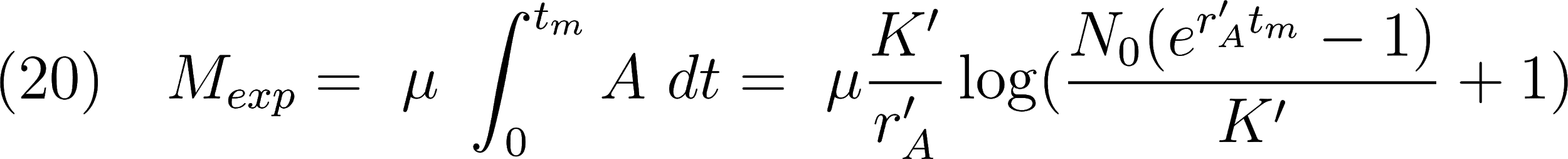
](https://www.codecogs.com/eqnedit.php?latex=(20)%20%5Cquad%20M_%7Bexp%7D%20%3D%20%5C%20%20%5Cmu%20%5C%20%20%5Cint_%7B0%7D%5E%7Bt_m%7DA%20%5C%20dt%3D%20%5C%20%5Cmu%20%20%5Cfrac%7BK'%7D%7Br_A'%7D%5Clog(%5Cfrac%7BN_0%20(e%5E%7Br_A'%20t_m%7D-1)%7D%7BK'%7D%2B1)%20#0)

Knowing the number of expected mutations, we can calculate the rescue probability, as the probability of having at least one mutation during the whole rescue time window:

[
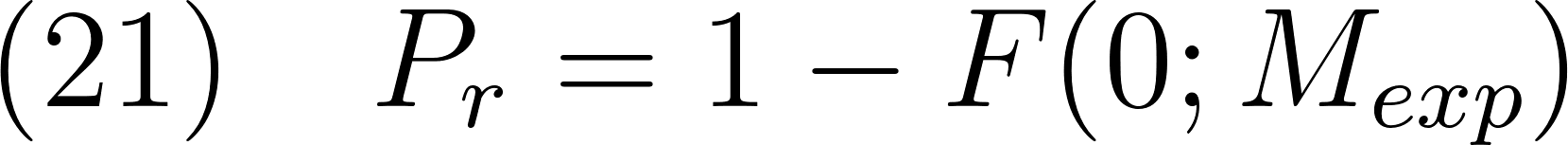
](https://www.codecogs.com/eqnedit.php?latex=(21)%20%5Cquad%20P_%7Br%7D%20%3D%201%20-%20F(0%3BM_%7Bexp%7D)#0)

Where [
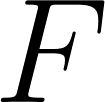
](https://www.codecogs.com/eqnedit.php?latex=F#0) is the cumulative distribution function of the Poisson distribution.

## Mutualism

Next, we formulated a theoretical analysis of evolutionary rescue probability in populations engaged in mutualism. In the case of mutualism, two mutation events are required for rescuing either of the species, and a different calculation of the expected number of mutations needs to be made both for the first mutation event [
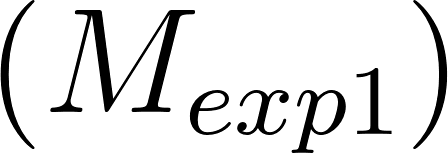
](https://www.codecogs.com/eqnedit.php?latex=(M_%7Bexp1%7D)#0), in which mutation can occur in both species, and the second one [
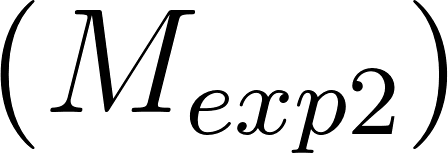
](https://www.codecogs.com/eqnedit.php?latex=(M_%7Bexp2%7D)#0) in which the remaining species has to adapt. We approximate the final rescue probability by calculating the expected number of mutations for these two events indpendantly and multiplying them. In the calculation of the second mutation, we assume that the first mutation occurs at the beginning of the rescue time window and that the second mutation can occur throughout the rescue time window. Thus, our approximation is expected to be overestimating the real value.

The first mutation event is calculated as for the intraspecies cooperation, with the exception of using a carrying capacity [
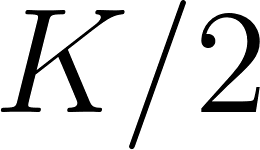
](https://www.codecogs.com/eqnedit.php?latex=K%2F2#0) due to the presence of the second species. Since both populations can adapt, and their behaviour is similar, the resulting probability for at least one mutation is multiplied by two.

The second mutation event can not be calculated the same way, since when the adapted mutants spread and approach the carrying capacity they outcompete their unadapted, ancestor partner species for resources. While the calculation of the rescue time interval ([
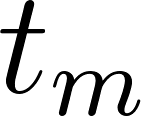
](https://www.codecogs.com/eqnedit.php?latex=t_m#0), eq.15) is similar, the change in the ancestor’s population size needs to be updated. This can be done by using a time-dependent carrying capacity, in which the limiting population size supportable by the environment changes through time due to environmental change. In our case, the carrying capacity of the unadapted species changes through time because of the increase in the population size of its adapted partner. Thus, the rate in change of carrying capacity is dependant on the rate of increase of mutant population, and can be described as:

[
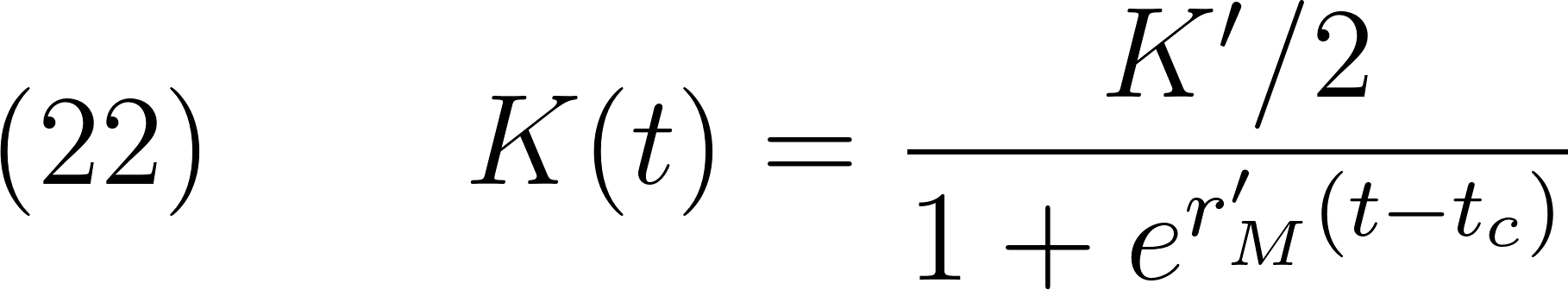
](https://www.codecogs.com/eqnedit.php?latex=(22)%20%5Cquad%20%5Cquad%20K(t)%20%3D%20%5Cfrac%7BK'%2F2%7D%7B1%2Be%5E%7Br_M'(t-t_c)%7D%7D#0)

Here, the carrying capacity has an inflection point [
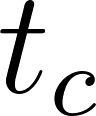
](https://www.codecogs.com/eqnedit.php?latex=t_c#0), that is the time at which the mutant reaches steady state:

[
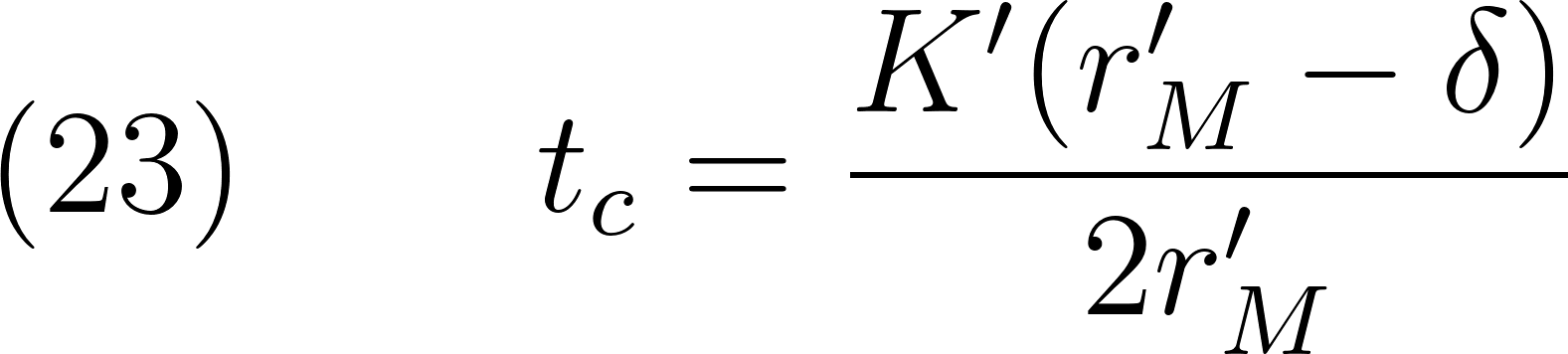
](https://www.codecogs.com/eqnedit.php?latex=(23)%20%5Cquad%20%5Cquad%20t_c%20%3D%20%5Cfrac%7BK'(r_M'-%5Cdelta)%7D%7B2r_M'%7D#0)

Thus, an approximation of the second species’ population density is given by:

[
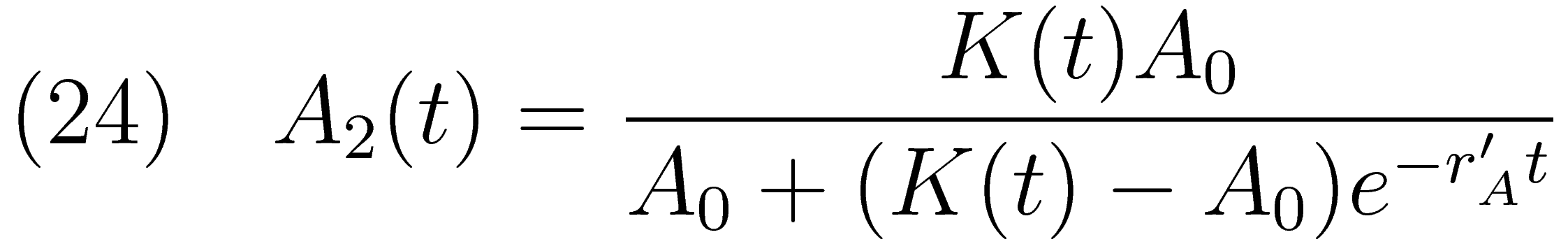
](http://www.sciweavers.org/tex2img.php?bc=Transparent&fc=Black&im=jpg&fs=100&ff=modern&edit=0&eq=(24)%20%5Cquad%20%5Cqaud%20A_2(t)%20%3D%20%5Cfrac%7BK(t)A_0%7D%7BA_0%2B(K(t)-A_0)e%5E%7B-r_A't%7D%7D#0)

And the expected number of the mutations in the second species can be calculated numerically:

[
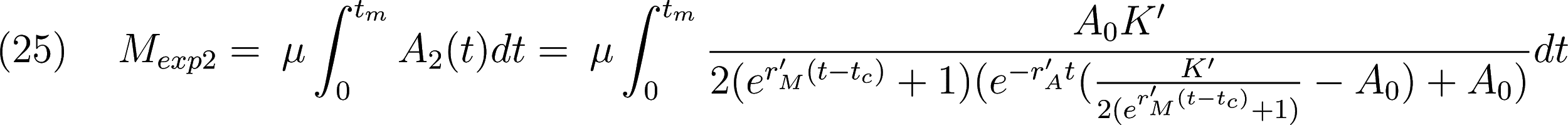
](https://www.codecogs.com/eqnedit.php?latex=(25)%20%5Cquad%20%5C%20M_%7Bexp2%7D%3D%20%5C%20%5Cmu%20%5Cint_0%5E%7Bt_m%7DA_2(t)dt%3D%5C%20%5Cmu%20%5Cint_0%5E%7Bt_m%7D%5Cfrac%7BA_0%20K'%7D%7B2%20(e%5E%7B%7Br_M'%7D%20(t-t_c)%7D%2B1)(e%5E%7B-%7Br_A'%7D%20t%7D(%5Cfrac%7BK'%7D%7B2(e%5E%7B%7Br_M'%7D%20(t-t_c)%7D%2B1)%7D-A_0)%2BA_0)%7Ddt#0)

Finally, the rescue probability can be approximated by multiplying the probabilities of at least one mutation occuring in each of the species during the rescue time window :

[
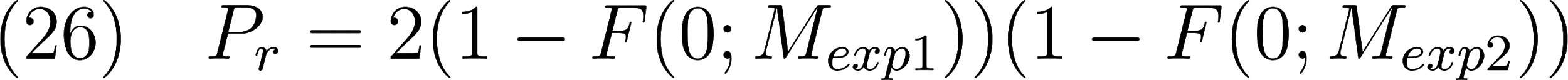
](https://www.codecogs.com/eqnedit.php?latex=(26)%20%5Cquad%20P_%7Br%7D%20%3D%202(1%20-%20F(0%3BM_%7Bexp1%7D))(1%20-%20F(0%3BM_%7Bexp2%7D))#0)

###

# Section D: Models with continuous Allee effect

## Intraspecies cooperation

We have constructed a model in which the Allee effect is implemented by a continuous function adapted from a previously published model^1^ (**Fig. S2)**. Here, the change in the ancestor and the mutant’s population size is similar to original model (**Eqs. 1-2)**, however the growth rate [
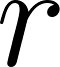
](https://www.codecogs.com/eqnedit.php?latex=r#0) is given by:

[
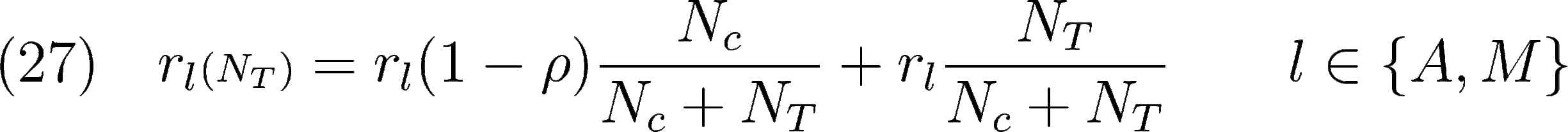
](http://www.sciweavers.org/tex2img.php?bc=Transparent&fc=Black&im=jpg&fs=100&ff=modern&edit=0&eq=(27)%20%5Cquad%20r_%7Bl%7D%7B%5Cscriptstyle%20(N_T)%7D%3D%20r_l(1-%5Crho)%5Cfrac%7BN_c%7D%7BN_c%2BN_T%7D%20%2Br_l%5Cfrac%7BN_T%7D%7BN_c%2BN_T%7D%20%5Cquad%20%5Cquad%20%7B%5Cscriptsize%20l%20%5Cin%20%5C%7BA%2CM%5C%7D%7D#0)

Thus, the transition between the two states of the growth rate function (growth rates at low and high cooperator density) is continuous.


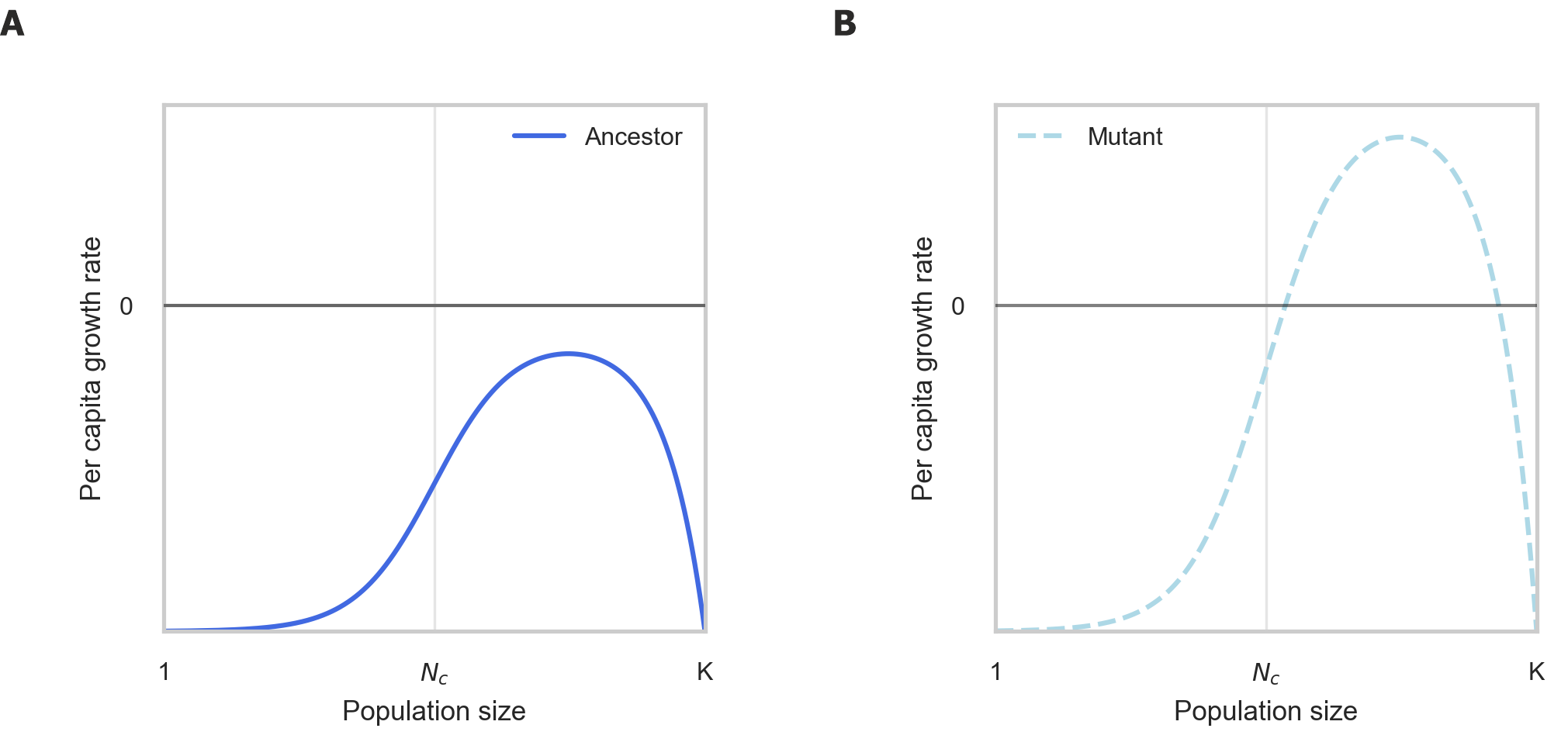


**Figure S2: Change in per capita growth rate of intraspecies cooperating population implemented with a continuous model.** (A) Ancestor per capita growth rate as a function of total population size after stress onset. When population size is above critical population size [
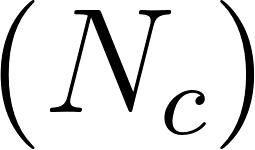
](https://www.codecogs.com/eqnedit.php?latex=(N_c)#0), growth rate decreases as population size increases due to carrying capacity (K). When below [
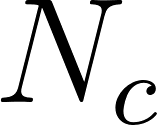
](https://www.codecogs.com/eqnedit.php?latex=N_c#0), growth rate reduces further due to Allee effect. Ancestor growth rate is always negative due to environmental stress. (B) Mutant per capita growth rate as a function of total population size after stress onset. Here, growth rate can be positive when above [
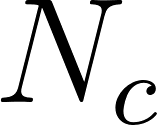
](https://www.codecogs.com/eqnedit.php?latex=N_c#0), thus adaptation is possible when total population size is sufficiently high.

Running simulations of this model with parameters similar to the ones used in the original model resulted in qualitatively similar results (**Fig. S3**). Quantitatively, the evolutionary rescue probability decreases with critical population size faster than in the discontinuous model.


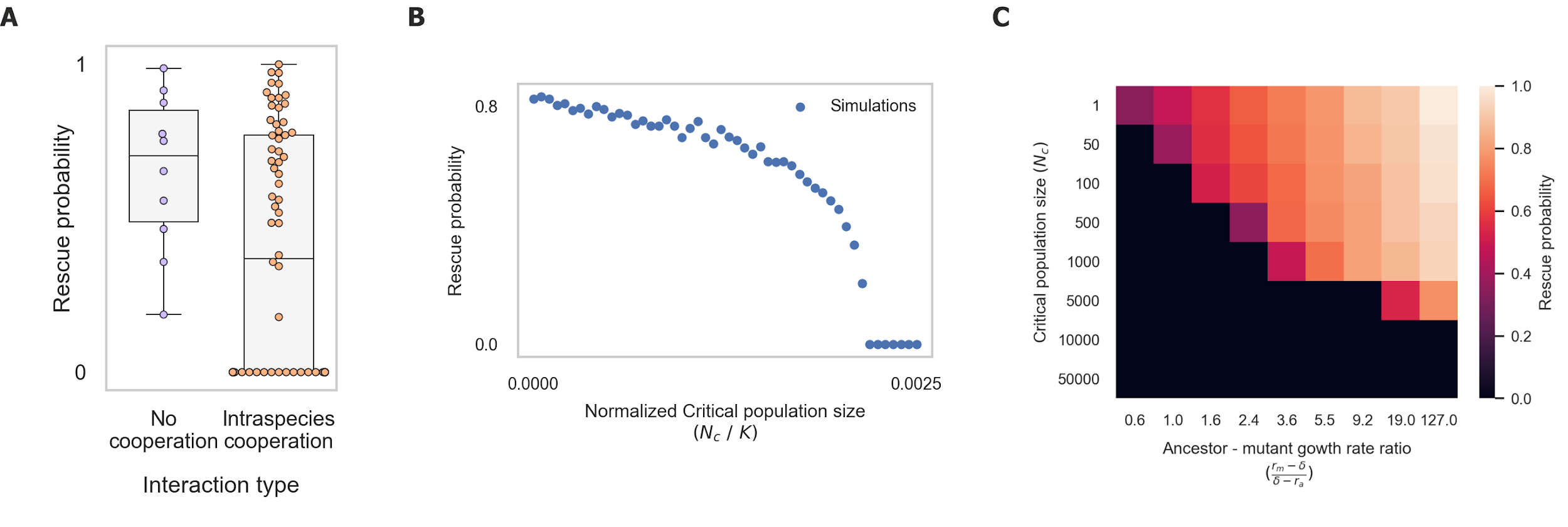


**Figure S3: The continuous model of cooperative populations shows qualitatively similar behaviour to that of the discontinuous model.** (A) Intraspecies cooperation has lower rescue probability in comparison to populations with no positive interactions, similarly to the discontinuous model. The median of the continuous model is lower since rescue probability decreases when critical populations size increases. Each dot represents the rescue probability resulted from 1000 simulations run with different set of parameters (Critical populations size (), ancestor and mutant’s growth rate ()). (B) The rescue probability decreases as the critical population size increases. (C) Rescue probability decreases with critical population size () and the ratio between mutant and ancestor growth rates.

## Mutualism

The implementation of continuous Allee effect of mutualistic interaction was also done by using the same description of the change in populations size (**Eqs. 6-7**), and altering the growth rate function:

Again, the transition between the two states of the growth rate function is continuous. Similar parameters to the original model were used and simulations resulted in qualitatively similar results (**Fig. S3)**. Once again, the evolutionary rescue probability decreases with critical population size faster than in the discontinuous model.

##

**Figure S4: The continuous model of mutualistic populations shows qualitatively similar behaviour to that of the discontinuous model.** (A) Rescue probability is greatly reduced in mutualisms compared to intraspecies cooperation, similarly to the discontinuous model. Dots represent the rescue probability calculated from simulations run with different sets of parameters as in figure S3. (B) Rescue probability decreases with critical population size () and the ratio between mutant and ancestor growth rates. (C) Death rate () and mutation rate () effect on evolutionary rescue.

## Cheaters - non-oscillatory model

In order to explore populations with a continuous change in population density of cooperative populations in the presence of cheaters, we implemented a previously published model^1^. Here, the growth rate functions of cooperators and cheaters are given by:

Simulations with varying  and  values showed that, as in the discontinuous model, evolutionary rescue is possible only in extreme cases in which cooperators have a growth advantage of orders of magnitude over the cheaters when at low density (**Fig.** **S5A**).

An important effect of the continuous model is that the dynamics observed between the cooperators and cheaters are not oscillatory (**Fig. S5B-C**). This behaviour can change the evolutionary rescue probability in comparison to the discontinuous model, since here both cooperator and cheater can adapt without a following collapse. Thus, cheaters are not necessarily purged from the populations. In addition, rescue probability can potentially be higher in extreme cases in comparison to the discontinuous model. However, our results show that group selection against populations with invaded cheaters is still strong when evolutionary rescue is required as apparent from the comparison to intraspecies cooperation.

1. Chen, A., Sanchez, A., Dai, L. & Gore, J. Dynamics of a producer-freeloader ecosystem on the brink of collapse. Nat. Commun. 5, 3713 (2014).

**Figure S5: Evolutionary rescue of cooperative populations in the presence of cheaters implemented by a continuous model is extremely unlikely .** (A) Rescue probability of cooperative population in the presence of cheaters is orders of magnitude lower than with no cheaters, similarly to the discontinuous model. Rescue in the continuous model was observed only in extreme cases, even more than the discontinuous model, in which cooperators have a growth advantage of orders of magnitude over the cheaters when at low density. (B+C) the dynamics observed between the cooperators and cheaters are not oscillatory. When  (B), rescue is not possible since cooperators density is below critical population size. Evolutionary rescue is only possible if  (A) when cooperators density prior to stress is above critical population size.

##

# Section E: Positive interactions provide a fitness advantage

## Comparison of non cooperating populations and interspecies cooperation

Populations engaged in intraspecies cooperation frequently benefit from a fitness advantage relative to non cooperating populations. Here, we implement this advantage by a higher growth rate of the intraspecies cooperating populations. Formally, both the ancestors and the mutant of the non cooperating populations have a decreased growth rate since they do not cooperate, which is set by a cost parameter (): :

Thus,  is the relative growth rate advantage of intraspecies cooperating population.

In order to compare the evolutionary rescue probability of non cooperating population with interspecies cooperation, we ran simulations of non cooperating populations with the updated model (**Eqs. S1-2,31**) with varying parameters. We have constructed a function of the rescue probability as a function of the cost value by interpolation, in order to test for what fitness advantage the rescue probability of non cooperating population and intraspecies cooperation is equal. Results show that for the relative growth rate advantage for which evolutionary rescue probability is equal to non cooperating population decreases with the critical population size (**Fig. S6**).

**Figure S6: Cooperative populations have a rescue probability comparable to that of non-cooperative ones only when their growth rate is significantly higher.** (A) Ratio of the growth rate of cooperating and non cooperating populations in which their evolutionary rescue probability is equal. At low critical populations size, the ratio is 1 since cooperation does not affect the evolutionary rescue probability. As critical population size increases, the ratio increases since the rescue time window decreases. At a high growth rate ratio, the evolutionary rescue matches only when both populations have no chance of rescue. (B) The same analysis for a wider parameters range. The ratio for which the evolutionary rescue probability of the two populations matches increases with the ratio between the growth rate of the mutant and ancestor, up to a point in which the growth rate of non cooperating species is twice that of non-cooperating populations for large critical population sizes.

## Interspecies cooperation and mutualism

Mutualistic interactions can provide fitness advantage through division of labor. Here, we compare the evolutionary rescue probability of populations engaged in intraspecies cooperation and those engaged in mutualism and benefit from an advantage in their growth rate. We use a cost parameter to describe the decreased growth rate of populations engaged in intraspecies cooperation:

We ran simulations of intraspecies cooperating populations with the updated model (**Eqs. S1-2,32**) with varying parameters. We again constructed a function of the rescue probability as a function of the cost value by interpolation, and compared the cost parameter for which evolutionary rescue probability of intraspecies cooperation is equal to the one for mutualism. We found that the growth rates of mutualistic populations must be greater by up to 30 percent in order for their rescue probability to be equal to that of cooperating ones (**Fig. S7**).

**Figure S7: Evolutionary rescue probability of mutualism and intraspecies cooperation equals when mutualism growth rate is significantly higher.** (A) Ratio of the growth rate of populations engaged in mutualism and interspecies cooperation in which their evolutionary rescue probability matches. As opposed to comparison with non cooperating populations, the ratio decreases with critical population size. At low critical population size, mutualisms must have a high growth rate advantage due to the requirement for two mutations and due to competition. At high critical population size, both rescue probabilities decrease to zero at the same critical population size due to equal limited rescue time window. (B) The same analysis for wider parameters range. The ratio for which the evolutionary rescue probability matches increases with the ratio between the growth rate of the mutant and ancestor.

##

# Section F: Model of mutualism in which populations are affected at high densities

In our model formulation, positive interactions affect a species’ exponential growth rate but not its carrying capacity (in the absence of the external death rate). This corresponds to a situation where the mutualism is important at low population densities, but at high densities growth is limited by another, independent factor. In order to ensure that our model choice did not have strong implications on the qualitative results observed in our work, we ran simulations with a modified model where the growth rate, rather than the change in population, is proportional to the size of the partner. In this model populations are affected by the mutualistic interactions at both low and high densities.

The rate of change in population size is given by:

Where . Running simulations of this model with parameters similar to the ones used in the original model resulted in qualitatively similar results (Fig, S11).

**Figure S11: Rescue probability of populations engaged in mutualism, where positive interactions affect populations at high densities, shows qualitatively similar results to that of the original model.** Dots represent the rescue probability calculated from simulations run with different sets of parameters as in figure 2 in the main text.

# **Table A**

| Parameter |  |  |  |  |  |  |  |  | Stress onset |  |  |  |
| --- | --- | --- | --- | --- | --- | --- | --- | --- | --- | --- | --- | --- |
| Description | Death rate | Ancestor growth rate | Mutant growth rate | Growth rate decrease in low densities | Mutation rate | Carrying capacity | Critical population size | Ancestor initial population density | Time point in which stress begins | Cheaters growth advantage at high cooperator density | Cooperators growth advantage at low densities | Decrease in growth rate |
| Units | $time^{-1}$ | $time^{-1}$ | $time^{-1}$ | $time^{-1}$ | $time^{-1}$ | $\#Individuals$ | $\#Individuals$ | $\#Individuals$ | $time$ | $time^{-1}$ | $time^{-1}$ | $time^{-1}$ |
| **No cooperation in** Figs. 1B, S8A | 1 | 0.909 | 1.4 | 0.612 | 10^-6 | 10^6 | 1 | 10^6 | 20 |  |  |  |
| **No cooperation**  **in** Figs:  2A, S3A, S6 | 1 | Within the range [0.6,0.99] | Within the range  [1,1.98]] | 1 | 10^-6 | 10^6 | 1 | 10^6 | 0 |  |  |  |
| **Intraspecies cooperation** in Figs:  2A, 2D, 3A, 4A, 5A, 6A,  S3A, S3C, S4A, S5A, S8B | 1 | Within the range [0.6,0.99 | Within the range  [1,1.98]] | 1 | 10^-6 | 10^6 | Within the range [1,500000] | 10^6 | 0 |  |  |  |
| **Intraspecies cooperation** in Figs:  2B,,2C,S8A | 1 | 0.909 | 1.615 | 0.612 | 10^-6 | 10^6 | 5000 | 10^6 | 10 |  |  |  |
| Fig. 2E | 1 | 0.909 | 1.615 | 1 | 10^-6 | 10^6 | Within the range [1,30000] | 10^6 | 0 |  |  |  |
| Fig. 2F | Within the range [0.8,  1.34] | 0.909 | 1.615 | 1 | lWithin the range [10^-8,  10^-3] | 10^6 | 1000 | 10^6 | 0 |  |  |  |
| **Mutualism in**  Figs:.  3A-C, 4A, 5A, 6A, S4A, S4B, S7, S11 | 1 | Within the range [0.6,0.99 | Within the range  [1,1.98]] | 1 | 10^-6 | 10^6 | Within the range [1,500000] | Both 5*10^5 | 0 |  |  |  |
| Figs. 4B-C | 1 | 0.909 | 1.615 | 0.612 | 10^-6 | 10^6 | 1000 | Both 5*10^5 | 10 |  |  |  |
| **Cheaters** in  Figs.  5A, 6A | InitialL:0.3  Stress: 1 | 0.909 | 1.615 | 0.612 | 10^-6 | 10^6 | 1000 | Random | 10 | Within the range [10^-2,1] | Within the range [10^-2,1] |  |
| Fig 5B | Initial : 0.3  Stress: 1 | 3 | 1.615 | 0.769 | 10^-6 | 10^6 | 1000 | Cooperator: 1000  Cheaters: 5*10^5 | 15 | 0.9 | 0.9 |  |
| Fig 5C-D | Initial : 0.5  Stress: 1 | 3 | 5.6 | 0.919 | 10^-6 | 10^6 | 1000 | Cooperator: 1000  Cheaters: 555555 | 15 | 0.9 | 0.009 |  |
| Fig. 5D | Initial : 0.5  Stress: 1 | 3 | 5.6 | 0.919 | 10^-6 | 10^6 | 1000 | Cooperator: 1000  Cheaters: 555555 | 15 | 0.9 | 0.009 |  |
| Fig S1A-B | 1 | 0.909 | 1.4 | 0.612 |  | 10^6 | 250,000 |  |  |  |  |  |
| Fig S2A-B | 1 | 0.909 | 1.4 | 1 |  | 10^6 | 1000 |  |  |  |  |  |
| **Fig. S3B** | 1 | Within the range [0.6,0.99 | Within the range  [1,1.98]] | 1 | 10^-6 | 10^6 | Within the range [1,500000] |  | 0 |  |  |  |
| Figs S4C, S9A | Within the range [0.8,1.34] | 0.909 | 1.615 | 1 | lWithin the range [10^-8,  10^-3] | 10^6 | Within the range [1,100000] | Both 5*10^5 | 0 |  |  |  |
| **Cheaters** in  Fig. S5A | Initial : 0.1  Stress: 1 | 0.909 | 1.615 | 0.612 | 10^-6 | 10^6 | 1000 | Random | 10 | Within the range [10^--3,1] | Within the range [10^-3,1] |  |
| **Fig S5B** | Initial : 0.3  Stress: 1 | 0.909 | 1.615 | 0.612 | 10^-6 | 10^6 | 1000 | Random | 10 | 0.2 | 1 |  |
| **Fig. S5C** | Initial : 0.3  Stress: 1 | 0.909 | 1.615 | 0.612 | 10^-6 | 10^6 | 1000 | Random | 10 | 1 | 0.2 |  |
| **Intraspecies cooperation** in Figs:  S6, S7 | 1 | Within the range [0.6,0.99 | Within the range  [1,1.98]] | 1 | 10^-6 | 10^6 | Within the range [1,500000] | 10^6 | 0 |  |  | Within the range [0,1] |
| **Fig. S9B** | 1 | 0.909 | 1.615 | 1 | 10^-6 | 10^6 | Within the range [1,30000] | Within the range [0,10^-6] | 0 |  |  |  |
| **Fig. S10** | 1 | 0.909 | 1.615 | 0.612 | 10^-6 | 10^6 | 500 | Both 5*10^5 | 10 |  |  |  |
| **Fig. S11** | 1 | Within the range [0.6,0.99 | Within the range  [1,1.98]] | 1 | 10^-6 | 10^6 | Within the range [1,500000] | Both 5*10^5 | 0 |  |  |  |
