# Supplementary figures and images for: Positive interactions within and between populations decrease the likelihood of evolutionary rescue
Source: PLoS Comput Biol. 2021 Feb 18;17(2):e1008732. doi: 10.1371/journal.pcbi.1008732 (PMC7924792; doi:10.1371/journal.pcbi.1008732)

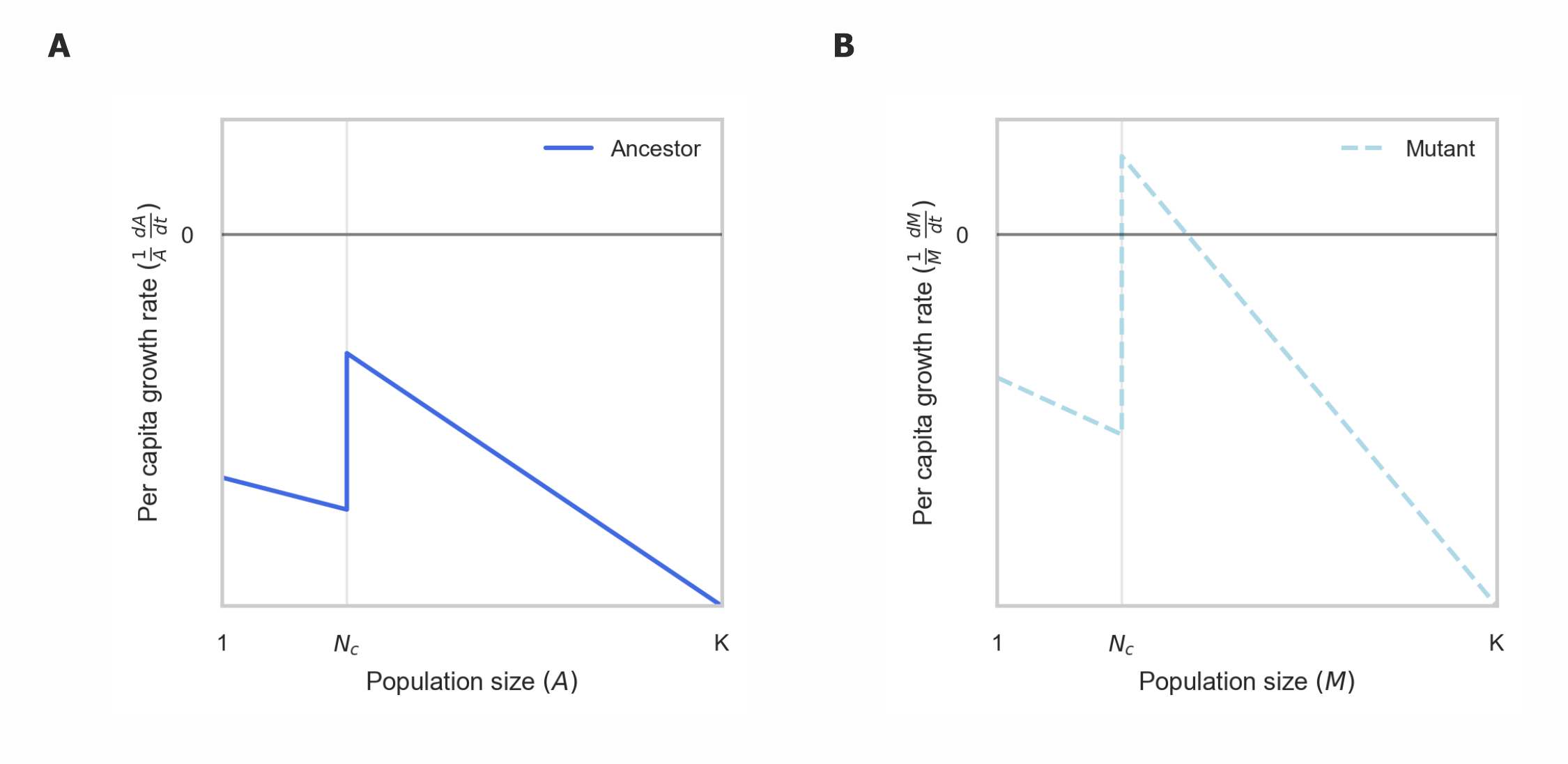

Supplement: S1 Fig — (A) Ancestor per capita growth rate as a function of total population size after stress onset. Growth rate decreases with population size due to intraspecies competition. When the population size is below the critical population size (NC), the growth rate reduces further due to the Allee effect. Ancestor growth rate is always negative due to environmental stress. (B) Mutant per capita growth rate as a function of total population size after stress onset. Here, growth rate can be positive above (NC) due to higher growth rate (rM) Thus, survival is possible when the total population size is sufficiently high. (TIF) [file pcbi.1008732.s002.tif]

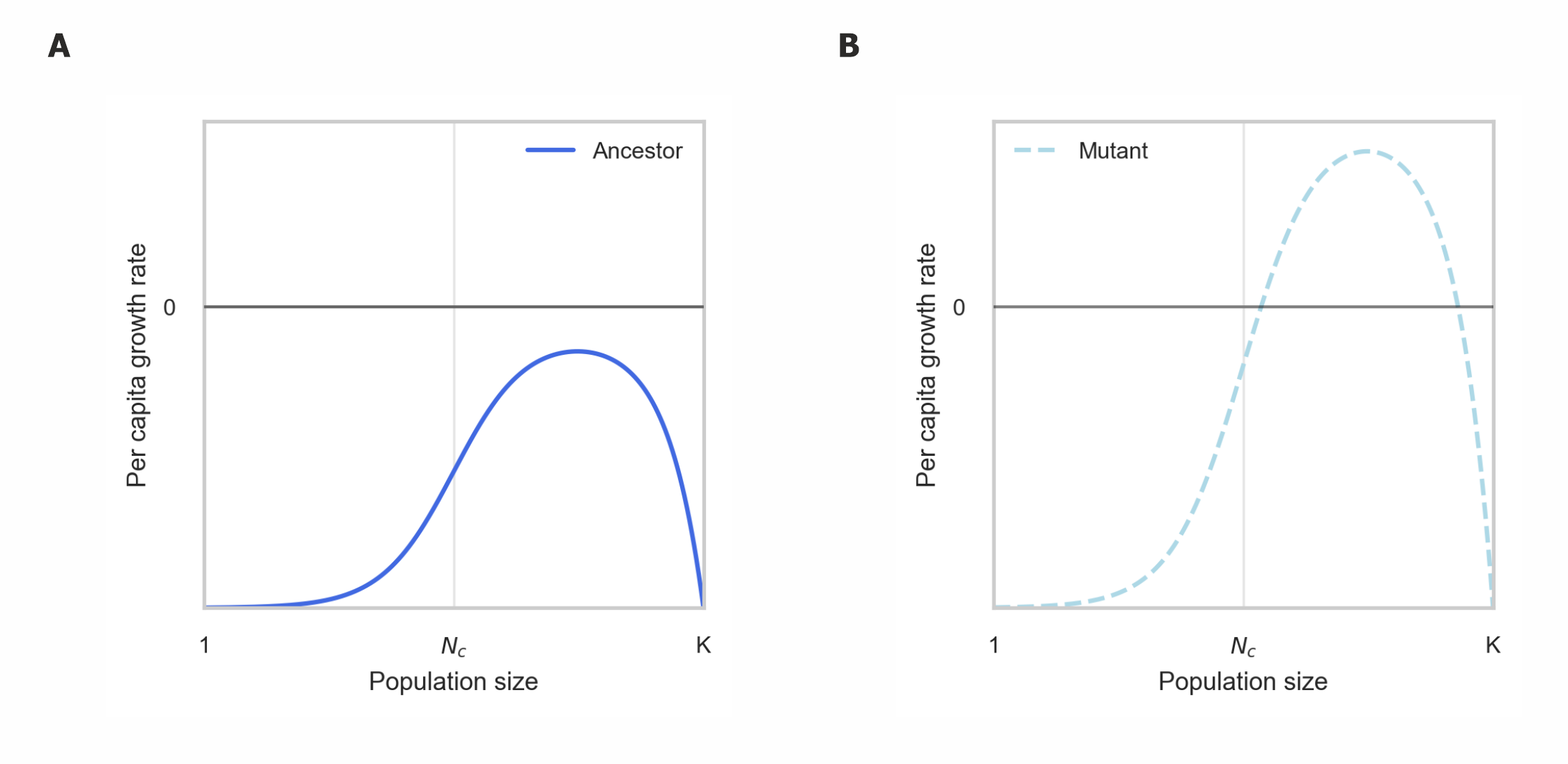

Supplement: S2 Fig — (A) Ancestor per capita growth rate as a function of total population size after stress onset. When population size is above critical population size (NC), growth rate decreases as population size increases due to carrying capacity (K). When below (NC), growth rate reduces further due to Allee effect. Ancestor growth rate is always negative due to environmental stress. (B) Mutant per capita growth rate as a function of total population size after stress onset. Here, growth rate can be positive when above (NC), thus adaptation is possible when total population size is sufficiently high. (TIF) [file pcbi.1008732.s003.tif]

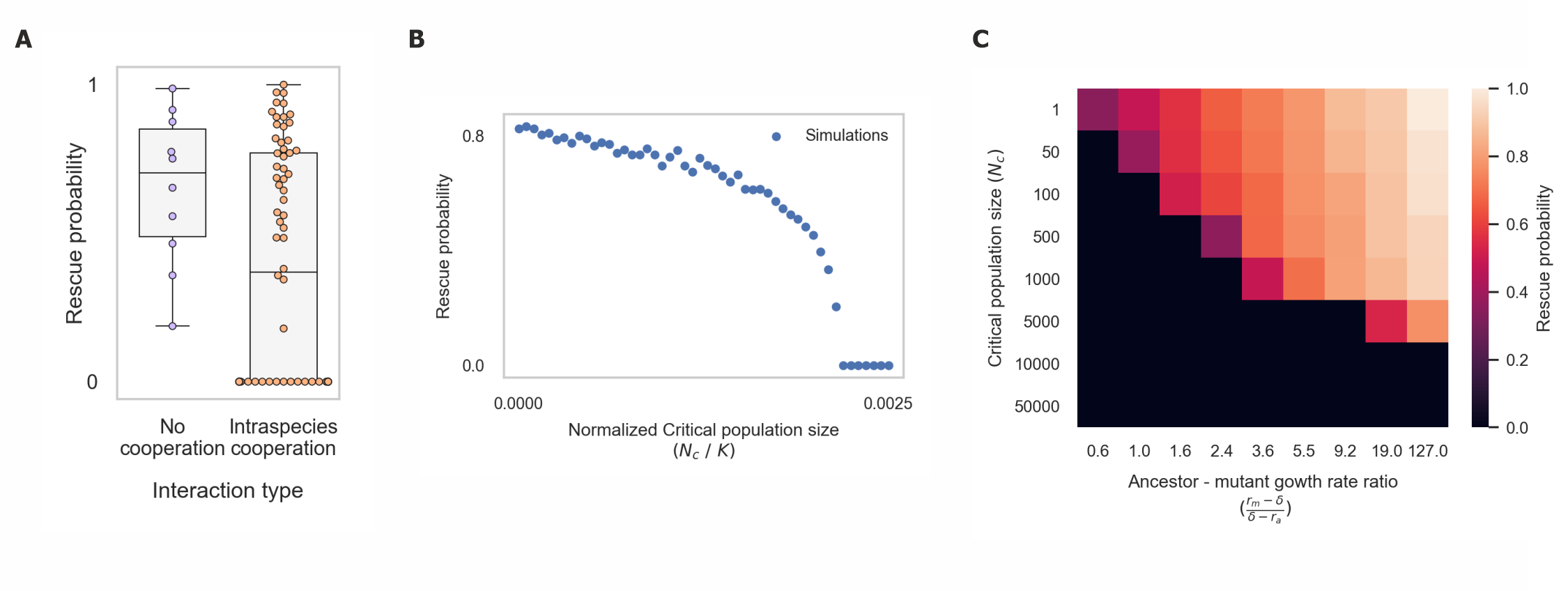

Supplement: S3 Fig — (A) Intraspecies cooperation has lower rescue probability in comparison to populations with no positive interactions, similarly to the discontinuous model. The median of the continuous model is lower since rescue probability decreases when critical populations size increases. Each dot represents the rescue probability resulted from 1000 simulations ran with different set of parameters (Critical populations size (NC), ancestor and mutant’s growth rate (rA,rM)). (B) The rescue probability decreases as the critical population size increases. (C) Rescue probability decreases with critical population size (NC), and the ratio between mutant and ancestor growth rates. (TIF) [file pcbi.1008732.s004.tif]

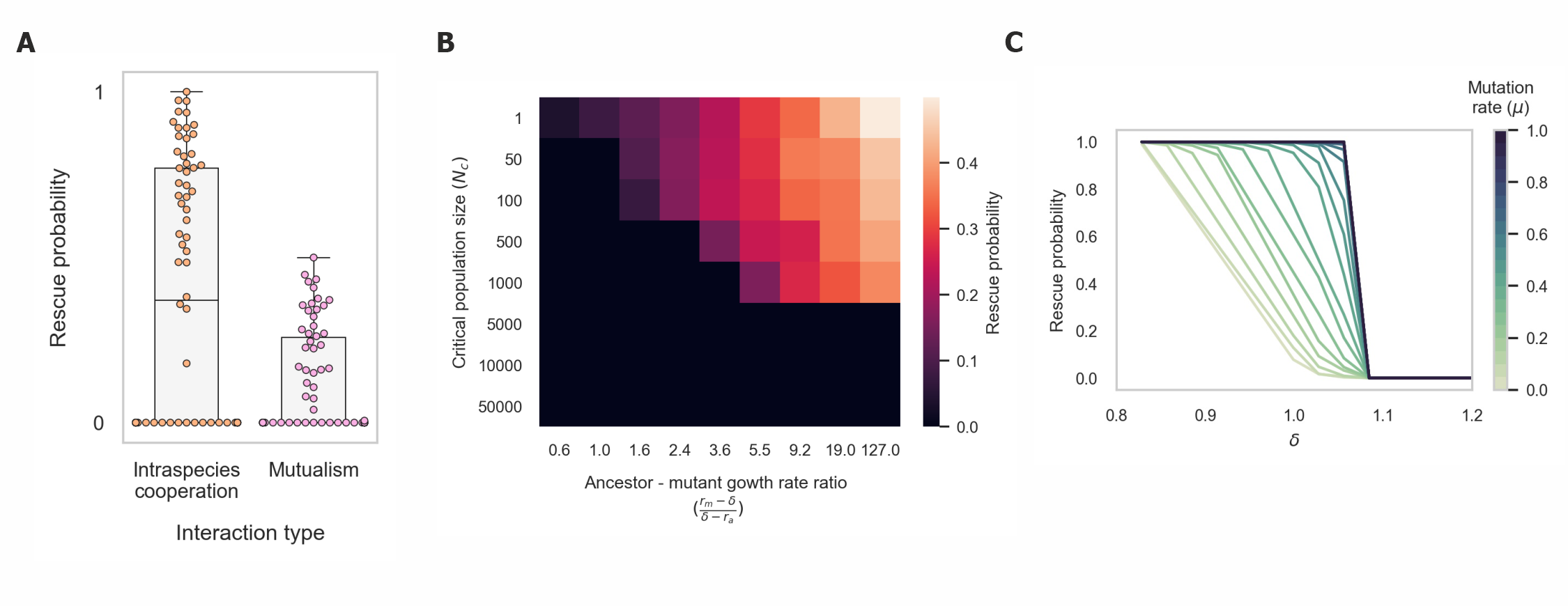

Supplement: S4 Fig — (A) Rescue probability is greatly reduced in mutualisms compared to intraspecies cooperation, similarly to the discontinuous model. Dots represent the rescue probability calculated from simulations ran with different sets of parameters as in S3 Fig. (B) Rescue probability decreases with critical population size (NC) and the ratio between mutant and ancestor growth rates. (C) Death rate (δ) and mutation rate (μ) effect on evolutionary rescue. (TIF) [file pcbi.1008732.s005.tif]

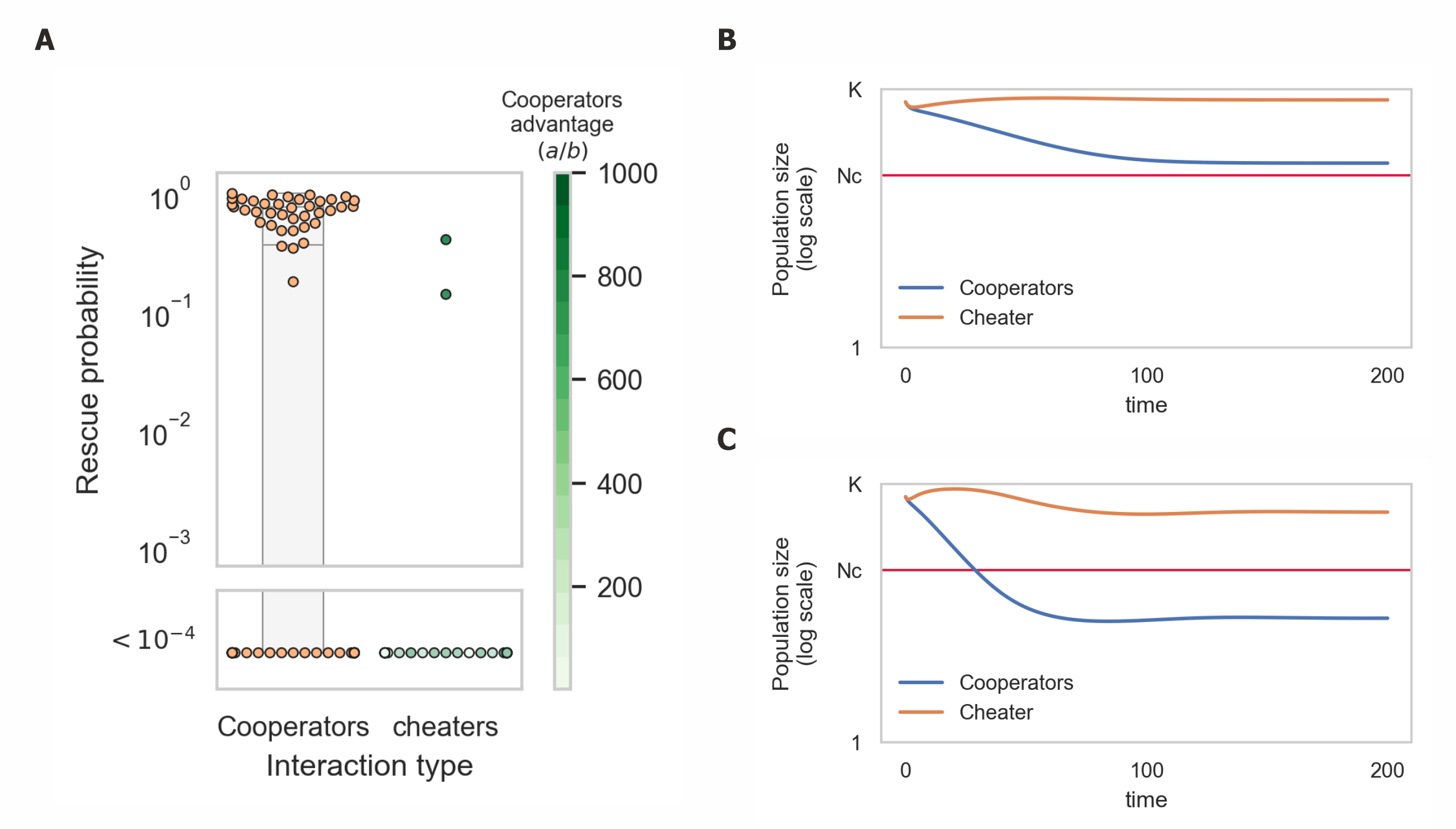

Supplement: S5 Fig — (A) Rescue probability of cooperative populations in the presence of cheaters is orders of magnitude lower than with no cheaters, similarly to the discontinuous model. Rescue in the continuous model was observed only in extreme cases, even more than the discontinuous model, in which cooperators have a growth advantage of orders of magnitude over the cheaters when at low density. (B+C) The dynamics observed between the cooperators and cheaters are not oscillatory. When b>>a (B), rescue is not possible since cooperators density is below critical population size. Evolutionary rescue is only possible if a>>b (A) when cooperators density prior to stress is above critical population size. (TIF) [file pcbi.1008732.s006.tif]

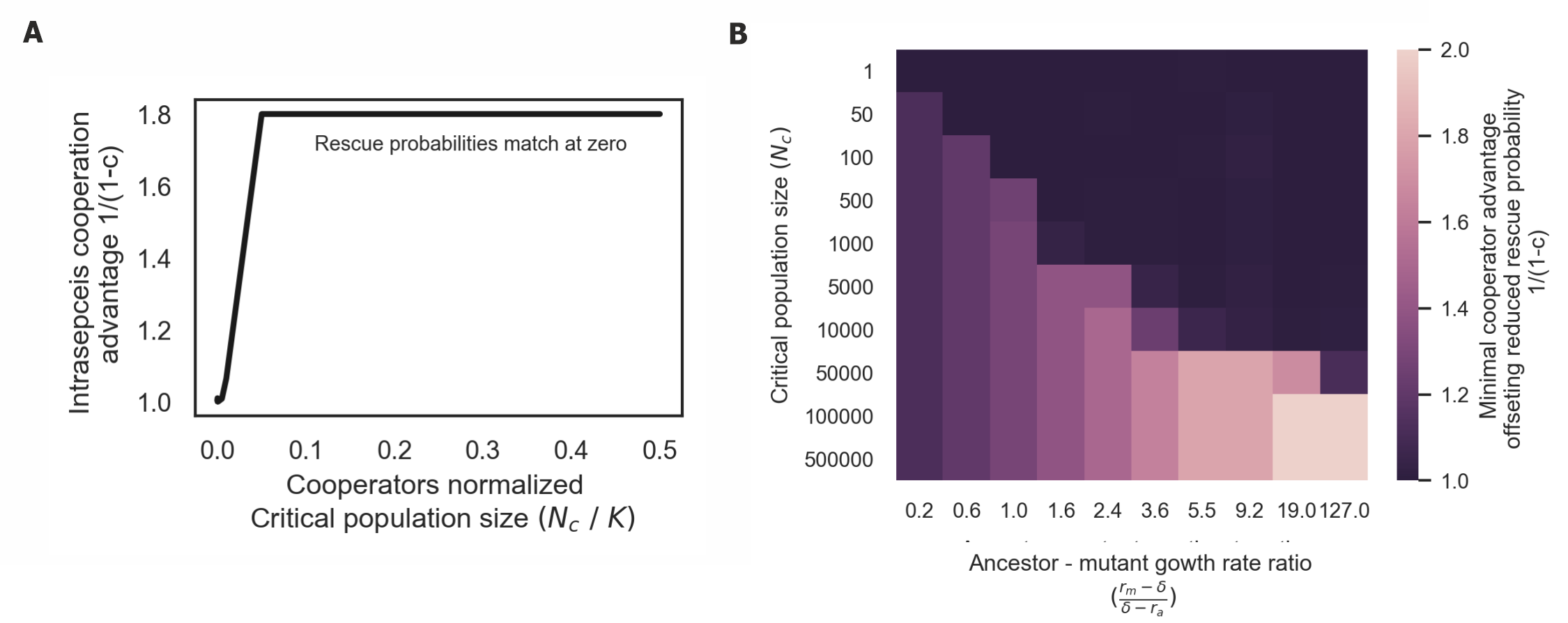

Supplement: S6 Fig — (A) Ratio of the growth rate of cooperating and non-cooperating populations in which their evolutionary rescue probability is equal. At low critical populations size, the ratio is 1 since cooperation does not affect the evolutionary rescue probability. As critical population size increases, the ratio increases since the rescue time window decreases. At a high growth rate ratio, the evolutionary rescue matches only when both populations have no chance of rescue. (B) The same analysis for a wider parameters range. The ratio for which the evolutionary rescue probability of the two populations matches increases with the ratio between the growth rate of the mutant and ancestor, up to a point in which the growth rate of non cooperating species is twice that of non-cooperating populations for large critical population sizes. (TIF) [file pcbi.1008732.s007.tif]

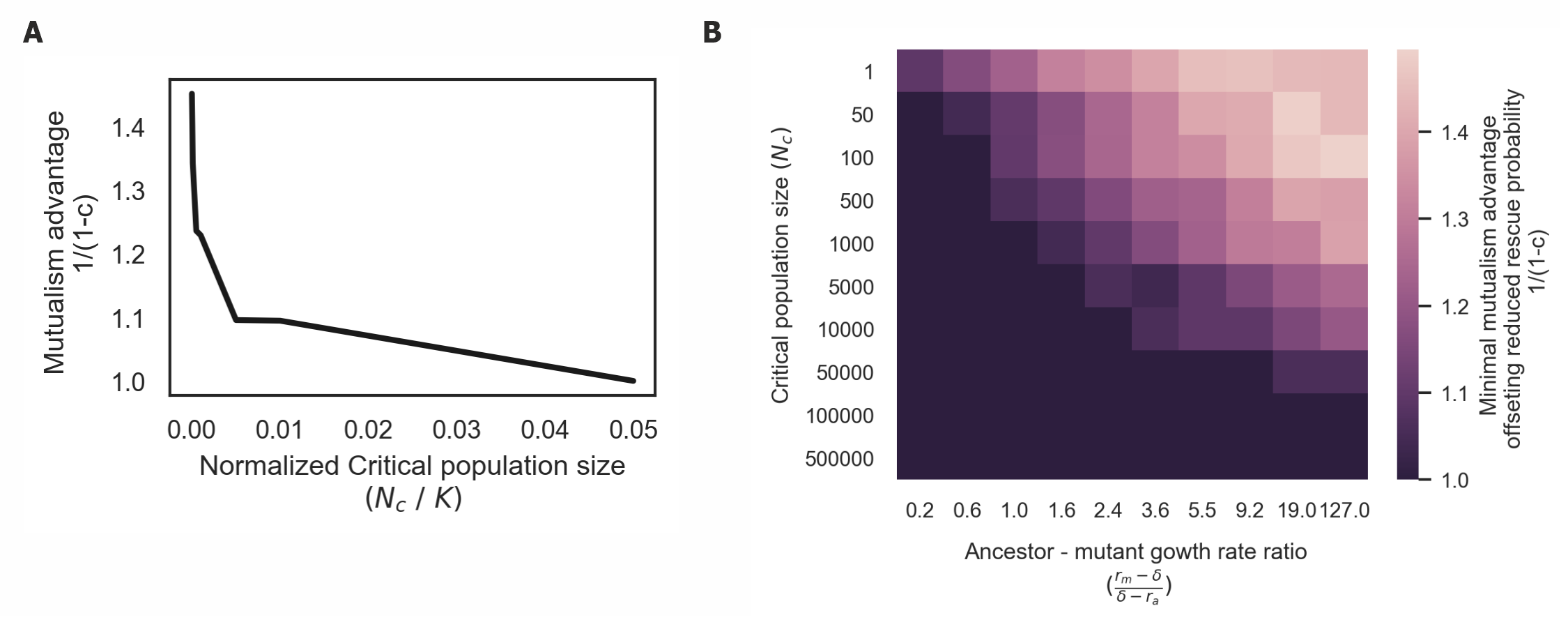

Supplement: S7 Fig — (A) Ratio of the growth rate of populations engaged in mutualism and interspecies cooperation in which their evolutionary rescue probability matches. As opposed to comparison with non-cooperating populations, the ratio decreases with critical population size. At low critical population size, mutualisms must have a high growth rate advantage due to the requirement for two mutations and due to competition. At high critical population size, both rescue probabilities decrease to zero at the same critical population size due to equal limited rescue time window. (B) The same analysis for wider parameters range. The ratio for which the evolutionary rescue probability matches increases with the ratio between the growth rate of the mutant and ancestor. (TIF) [file pcbi.1008732.s008.tif]

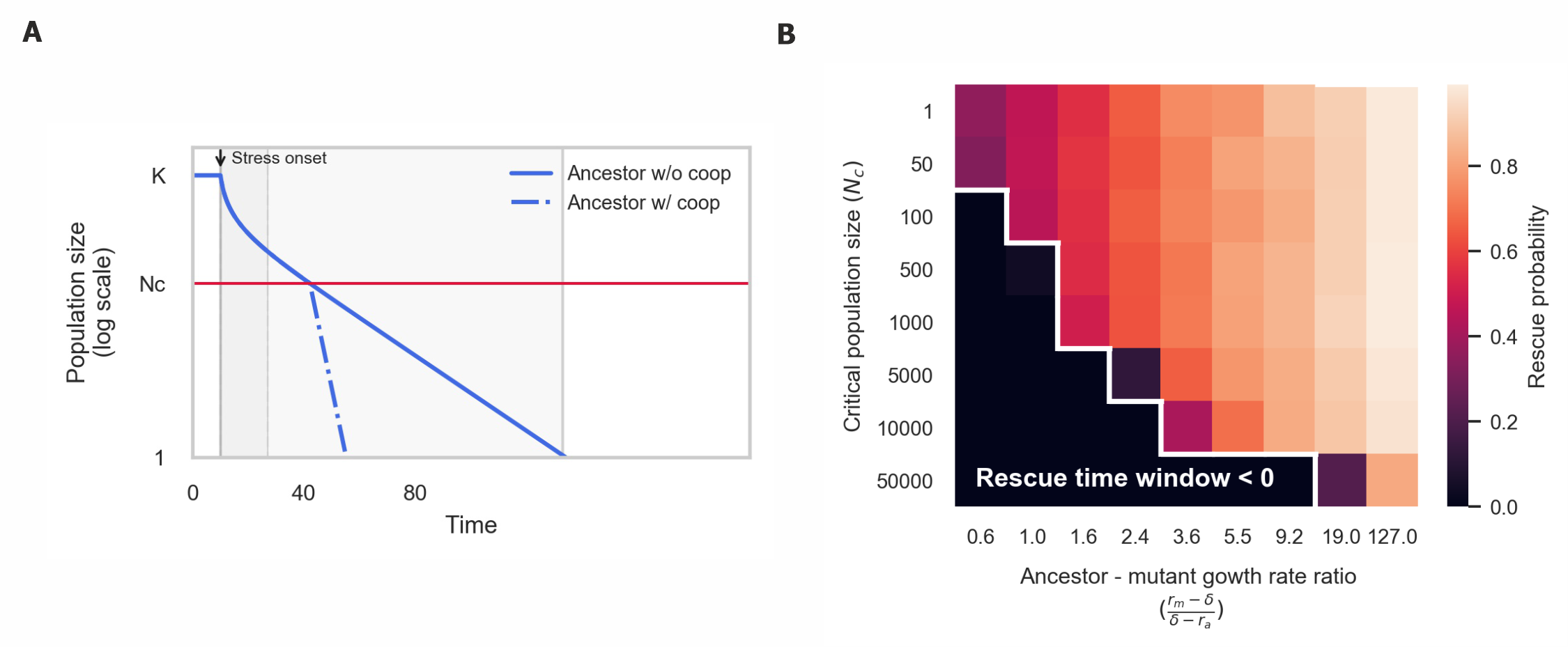

Supplement: S8 Fig — (A) Comparison of the rescue time window (grey) of non cooperating populations (solid line, light grey) and populations engaged with interspecies cooperation (dashed line, dark grey). (B) Rescue probability decreases with critical population size (NC) and the ratio between mutant and ancestor growth rates. The theoretical rescue time window (white line) reveals a transition curve under which the rescue probability is zero. (TIF) [file pcbi.1008732.s009.tif]

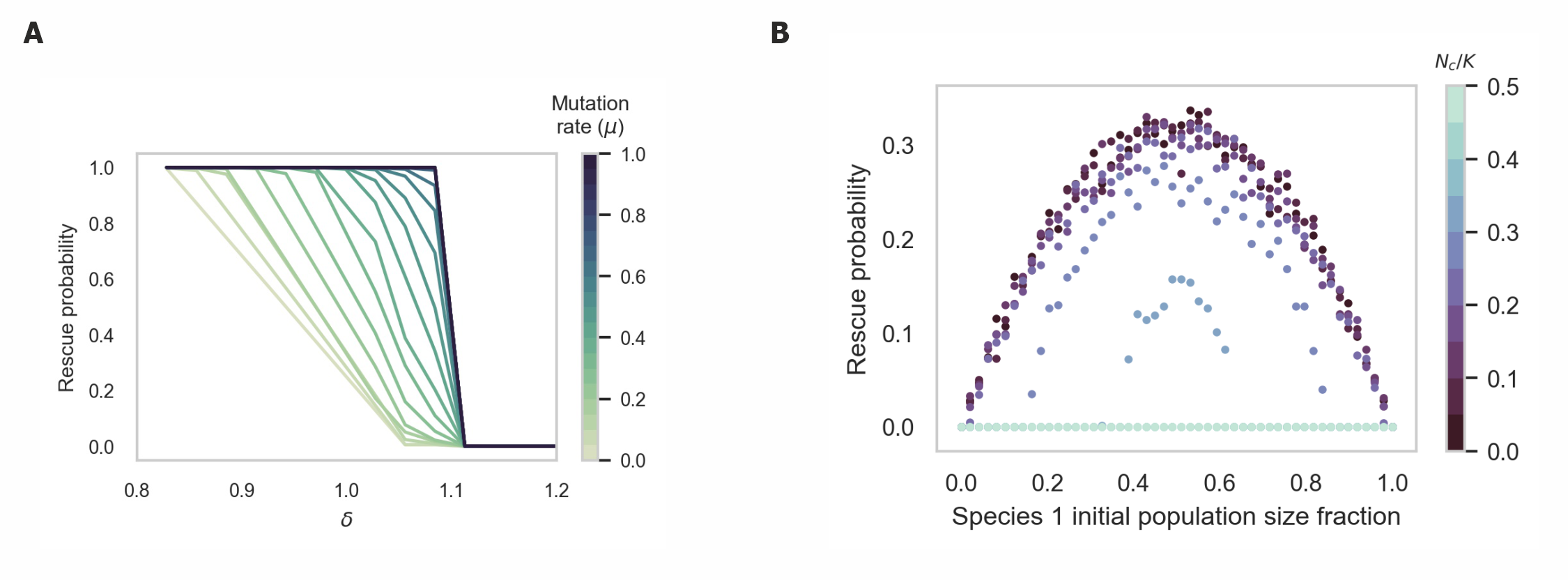

Supplement: S9 Fig — (A) Death rate (δ) and mutation rate (μ) effect on evolutionary rescue probability of mutualistic populations. (B) Evolutionary rescue probability as a function of the fraction of initial population size of one species. Rescue probability decreases as one of the mutualistic partners begins at lower initial density. (TIF) [file pcbi.1008732.s010.tif]

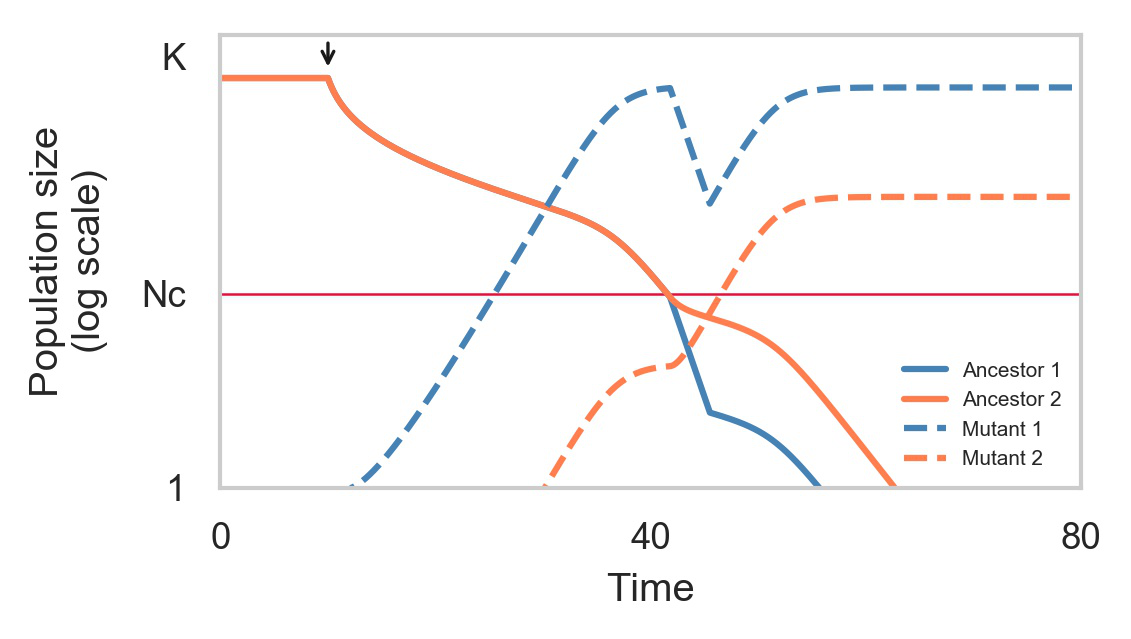

Supplement: S10 Fig — In limited conditions, adaptation of one of the species can increase the rescue time window of its partner. An example of simulation in which adaptation of one of the species (blue) increases the rescue time window of its partner (orange). Since the adapted mutant spreads and reaches critical population size, the growth rate of its cooperator is not impaired. Thus, the second mutation event can happen at any time point prior to the ancestors’ extinction. (TIF) [file pcbi.1008732.s011.tif]

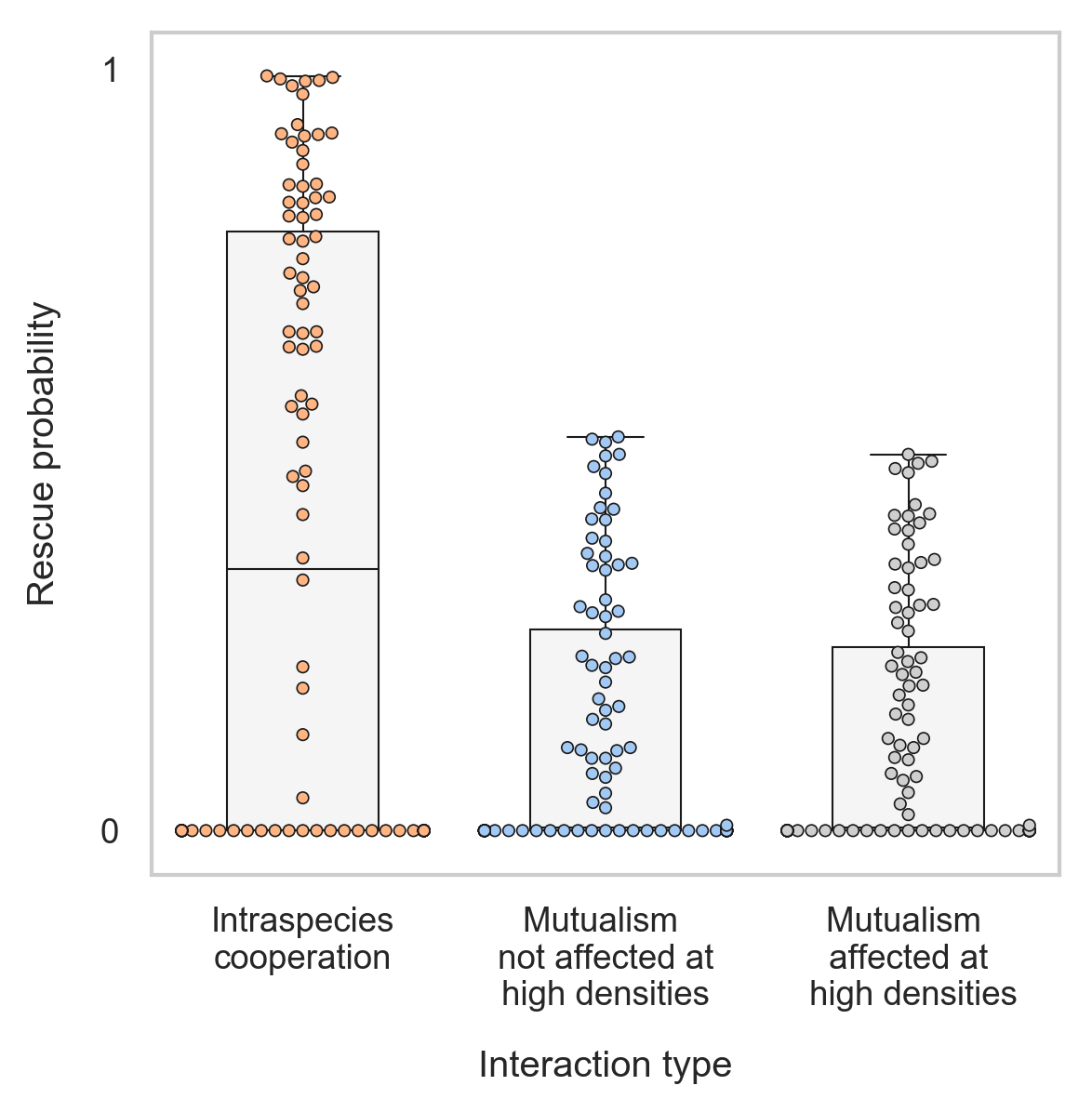

Supplement: S11 Fig — Dots represent the rescue probability calculated from simulations ran with different sets of parameters as in Fig 2 in the main text. (TIF) [file pcbi.1008732.s012.tif]
