# Supplementary material for: Positive interactions within and between populations decrease the likelihood of evolutionary rescue
Source: PLoS Comput Biol. 2021 Feb 18;17(2):e1008732. doi: 10.1371/journal.pcbi.1008732 (PMC7924792; doi:10.1371/journal.pcbi.1008732)
Supplement: Attachment — Submitted filename: Response_letter.docx [file pcbi.1008732.s013.docx]

**Thank you for your consideration of our manuscript. We are encouraged by the positive response of the reviewers, and we appreciate their constructive comments. These are our detailed responses to each of the reviewers’ comments:**

Reviewer #1

The authors set up an in silico investigation of the influence of positive interactions on a populations chance of rescue in a harsh environment. The model is based on a logistic growth, modified to incorporate the Allee effect and environment-dependent death rate. The rescue assay they have set up is also intuitive and suitable for the focal question. Overall, the paper is well-written, has a logical flow, and is accessible in my opinion to a broad audience.

In my view, the paper adds to the current understanding of the communities at the intersection of ecology and evolution and would be suitable for publication after addressing the following comments:

1. The main findings of the paper appear to be resulting from enforcing the Allee effect on small populations, which disproportionately impacts positive interactions. It is possible that positive interactions do not follow this model, especially in a structured environment (see for example (Chao & Levin, PNAS, 1981) for intraspecies cooperation or (Momeni et al, eLife, 2013) for interspecies cooperation)

**Population size indeed has a strong impact on species’ ability to adapt to new environments. We have focused on well-mixed populations, rather than ones occupying a structured environment. In this scenario, interaction occurs between all individuals in the population. We have primarily worked with fairly large population sizes (carrying capacity K ~10^6^, Table S1), thus the number of interacting individuals in our simulations is typically large. The rescue probability of populations engaged in positive interactions are also highly dependent on the value of critical population size, which can be represented as the fraction of the carrying capacity. Thus, in our work an emphasis was made on understanding the impact of the ratio between the critical population size and the population density on the rescue probability (Fig. 2E).**

**We completely agree that in structured environments the effect of positive interactions on evolutionary rescue dynamics can be more complex. We have edited the discussion to point out this important point (line 402):**

***“Lastly, spatial structure,where individuals can interact preferentially with their neighbors and kin has been shown to promote positive interactions and may increase the likelihood of evolutionary rescue***[***^1–3^***](https://www.zotero.org/google-docs/?V3Nn3u)***.”***

2. I have concerns about Eq. S6 and S7. In mutualism, conventionally the growth rate of each species is set to be modulated by the population size of the other species. However, in the current formulation, the rate of change in populations is proportional to the population size of the partner. The current formulation would perhaps make sense if the mutualism is based on exchange of consumable mediators. I think the justification for this formulation should be included in the manuscript. Also, if not overly burdensome, I recommend confirming the results using a Lotka-Volterra type formulation as well.

**We thank the reviewer for pointing this issue and stressing the need for clarification of our models choice. The following clarification was added to the model description in the main text (line 222):**

***“In our model formulation, positive interactions affect a species’ exponential growth rate but not its carrying capacity (in the absence of the external death rate). This corresponds to a situation where the mutualism is important at low population densities, but at high densities growth is limited by another, independent factor. For example, when species provide each other with essential amino acids or cross-protection from antibiotics while still competing for another limited resource, such as a carbon source. Such formulation has been used in several recent works, where it agreed well with experimental results***[***^4–6^***](https://www.zotero.org/google-docs/?E4gZ2E)***. “***

**In order to ensure that our model choice did not have strong implications on the qualitative results observed in our work, we followed the suggestion in the comment and ran simulations with a modified model where the growth rate, rather than the change in population, is proportional to the size of the partner. In this model populations are affected by the mutualistic interactions at both low and high densities. The following model and figure (Fig S11) was added to section 6 in the supplementary materials (Line 430):**

***“The rate of change in population size is given by:***

<https://www.codecogs.com/eqnedit.php?latex=(1)%20%5Cquad%20%5Cquad%20%20%5Cfrac%7BdA_i%7D%7Bdt%7D%20%3D%20A_i%20%20(r_%7BAi%7D%7B%5Cscriptstyle%20(N_%7BTj%7D)%20%7D%20-%20c_%7BAi%7D%20N_i-c_%7BAi%7DN_j)%20-%20%5Cdelta%20A_i#0>

<https://www.codecogs.com/eqnedit.php?latex=(2)%20%5Cquad%20%5Cquad%20%20%5Cfrac%7BdM_i%7D%7Bdt%7D%20%3D%20%20M_i%20%20(r_%7BMi%7D%7B%5Cscriptstyle%20(N_T_j)%20%7D-c_%7BMi%7D%20N_i-c_%7BMi%7DN_j)%20-%20%5Cdelta%20M_i#0>

***Where***<http://www.sciweavers.org/tex2img.php?bc=Transparent&fc=Black&im=jpg&fs=100&ff=modern&edit=0&eq=c_%7Bli%7D%20%3D%20r_%7Bli%7D%2FK%20%2C%20%5C%20l%20%5Cin%20%5C%7BN%2CM%5C%7D#0>***. Running simulations of this model with parameters similar to the ones used in the original model resulted in qualitatively similar results (Fig. S11).***

**Figure S11: Rescue probability of populations engaged in mutualism, where positive interactions also affect populations at high densities, shows qualitatively similar results to that of the original model.** Dots represent the rescue probability calculated from simulations run with different sets of parameters as in figure 2 in the main text. **“**

3. In mutualism results, the assumption is that the environmental stress affects both populations. This makes it somewhat trivial that the rescue is less likely for mutualism compared to a monoculture rescue. It would be interesting to test what happens if the stress affects only one of the populations. In other words, is the rescue less or more likely for a species in an abiotic environment versus in a biotic environment of its mutualistic partner.

**This is an interesting scenario that we had not considered previously. Following the comment, we ran simulations of the scenario suggested, in which only a single species is affected by the environmental stress and needs to adapt to the new environment. We found that, in this scenario, the likelihood of evolutionary rescue depends strongly on the degree of competition between the species. In mutualistic populations that also compete strongly with each other rescue is not possible, since the adapted mutant can not invade the unaffected species population (Fig. R1A). This result is independent of the growth rates of the species, and can be shown analytically. A detailed analysis is presented below.**

**However, when there is no competition between the species, rescue probability is higher than that of intraspecies cooperation, but worse than that of non cooperating populations (Fig. R1B). This might be because the unaffected species manages to get to its carrying capacity, making the rescue time window longer. Since this analysis only begins exploring the complexities of this scenario, including the effects of the strength and symmetry of interactions, we have decided not to include it in the current manuscript, and defer it to future work.**

**Figure R1: Rescue probability of populations engaged with mutualism, where only single species is affected by external stress.** (A) An example of simulation in which the partners compete. The species that is affected by the environmental stress (Blue) manage to adapt, but eventually gets extinct due to competition with the unaffected species (Orange). Thus, if the two species compete, rescue is not possible. (B) When there is no competition between the two species, rescue probability is higher than that of intraspecies cooperation, but worse than that of non cooperating populations

**Analysis of the dynamics of a mutualistic interaction between a species that is not affected by an external stress, and a mutant that is affected:**

**Let us consider the case where the species affected by the stress (species 1) has already adapted, and model the competition between its adapted population (M1) and that of the other, unaffected species that has not evolved (A2). The population dynamics in this case are given by:**

<https://www.codecogs.com/eqnedit.php?latex=(1)%20%5Cquad%20%5Cquad%20%20%5Cfrac%7BdM_1%7D%7Bdt%7D%20%3D%20r_%7BM1%7D%7B%5Cscriptstyle%20(N_%7BT2%7D)%20%7D%20%20M_1%20%20(1-%5Cfrac%7BM_1%2B%5Calpha_%7B12%7DA_2%7D%7BK%7D)%20-%20%5Cdelta%20M_1#0><https://www.codecogs.com/eqnedit.php?latex=(2)%20%5Cquad%20%5Cquad%20%20%5Cfrac%7BdA_2%7D%7Bdt%7D%20%3D%20r_%7BA2%7D%7B%5Cscriptstyle%20(N_%7BT1%7D)%20%7D%20%20A_2%20%20(1-%5Cfrac%7BA_2%2B%5Calpha_%7B21%7DM_1%7D%7BK%7D)#0>

**where** [**represents**](https://www.codecogs.com/eqnedit.php?latex=%5Calpha_%7Bji%7D#0) **the competitive effect coefficients of species**<https://www.codecogs.com/eqnedit.php?latex=i#0> **on species**<https://www.codecogs.com/eqnedit.php?latex=j#0>**.**

**As previously shown**[**^8^**](https://www.zotero.org/google-docs/?YxNAiX)**, the external death rate (**$\delta$**) can be integrated into the competition coefficients, resulting in the new coefficients:**

<https://www.codecogs.com/eqnedit.php?latex=(3)%20%5Cquad%20%5Cquad%20%5Cwidetilde%7B%5Calpha%7D_%7B12%7D%3D%5Calpha_%7B12%7D%5Cfrac%7B1%7D%7B1-%5Cfrac%7B%5Cdelta%7D%7Br_%7BM1%7D%7D%7D#0>

<https://www.codecogs.com/eqnedit.php?latex=(4)%20%5Cquad%20%5Cquad%20%5Cwidetilde%7B%5Calpha%7D_%7B21%7D%3D%5Calpha_%7B21%7D(1-%5Cfrac%7B%5Cdelta%7D%7Br_%7BM1%7D%7D)#0>

**Now, since in our case**<https://www.codecogs.com/eqnedit.php?latex=%5Calpha_%7B12%7D%3D%5Calpha_%7B21%7D%3D1#0>**, and** $r_{M1}> \delta$ **we get that**<https://www.codecogs.com/eqnedit.php?latex=%5Cwidetilde%7B%5Calpha%7D_%7B12%7D%3E1#0> **and**<https://www.codecogs.com/eqnedit.php?latex=%5Cwidetilde%7B%5Calpha%7D_%7B21%7D%3C1#0>**. Hence, species 2 is a stronger competitor and species 1 will go extinct.**

4. In studying the impact of cheaters, the authors are focusing on a situation that the stable population ratio of the community is heavily in favor of the cheater. Do you expect the same results if the stable community composition contained a cooperator majority and a minority of cheaters?

**The fact that, at steady state, the ratio of the population is in favor of the cheaters is not a result of a choice we made, but a result of the population dynamics. It was previously shown that in cooperative populations invaded by cheaters, the density of the cooperators decreases toward the population critical size**[**^9^**](https://www.zotero.org/google-docs/?tSKEqB)**. Intuitively, cheaters have an advantage over cooperators when the cooperator population is above the critical density (due to not paying the cost of cooperation), and a disadvantage when cooperators are below critical size (e.g. due to cooperators’ partial privatization of public goods). Therefore, in a steady state with cheaters, cooperator population size tends to be maintained around their critical size. Note that this occurs both when there is a sharp transition in growth rate at the critical cooperator population size, and when the dependence is more gradual (Fig. S5). How close the cooperator population is to the critical size is determined by the ratio between the cooperators’ advantages at low densities (a) and the cheaters’ advantage at high densities (b). Thus, in figure 5A we test a wide range of this parameter. Indeed, rescue was observed only when the cheater's advantage is much higher than the one of the cooperators (b>>a), a scenario in which the cheater's density prior to stress onset is relatively low. We have added a clarification of this important point to the main text (Line 343):**

***“Intuitively, this happens since above the critical population size cheaters, who do not pay the cost of cooperation, have a growth advantage, whereas cooperators are at an advantage below the critical population size (e.g. due to preferential access to the public goods).”***

Reviewer #2:

To understand the effects of positive interactions on the ability to adapt to a stress, Goldberg and Friedman performed numeric simulations mimicking microbial growth in a flask (no spatial structure). The authors modeled two types of positive interactions: intraspecies cooperation and interspecies mutualism. First, they modeled cooperation by modifying the logistic growth model to include the Allee effect which caused the growth rate to slow below a critical population threshold (Nc). To avoid extinction it was critical that the population remained above the Nc. At the beginning of the simulations, an ancestral population was exposed to a stress that causes them to rapidly die (the death rate exceeded the growth rate). During this period, mutants were modelled to arise as a Poisson process. These mutants had an increased growth rate and could therefore survive the stress if they reached a population size above the Nc. The mutation rate and ancestral population size affected the likelihood of mutants arising at any given time. These mutants were dependent on the population remaining above the Nc or else they too would experience the Allee affect and go extinct. Not surprisingly, the authors found that non-cooperators -- populations that did not experience the Alee effect -- had a higher probability of survival than the cooperator populations. When the populations (both cooperative and non-cooperative) survived the stress it was due to mutants arising within the ‘rescue time window’. Secondly, the authors modeled mutualism in which a species’ growth rate was dependent on the other species’ population remaining above the critical population size in order to avoid the Allee effect. The authors found that under mutualistic conditions, species had a higher probability of extinction than under a cooperative scenario because mutants in both species needed to arise within the rescue time window. Unsurprisingly, cheaters and competition further reduced the probability of stress survival.

Overall, this paper advances the discipline and sets the stage for future experimental work.

Major Comments: NA

Minor Comments:

Throughout the manuscript there are many typographical errors. In figure 2A, “parameter” is misspelled. In figure 3C, “Ancestor” is misspelled. Furthermore, there are many additional typos in the supplementary material and code repository. Please proofread and correct these mistakes..

**Thank you for drawing our attention to these mistakes. We have proofread the manuscript and corrected such mistakes. FIgures in which typographical errors were fixed are highlighted in yellow in the tracked-changes version of the manuscript.**

Figure 2E was initially confusing to me. Please include a label for the ‘Rescue time window’ in the symbol key in the top right corner.

**We have updated the figure. We appreciate the helpful note since we try to make our figures as clear as possible.**

Lines 122-124: Provide a citation for this statement.

**We have added a citation for this statement, thank you for the comment.**

Lines 248-252: Provide the equation #s that describes competition for resources in the mutualism.

**Competition for resources in mutualism is captured by the fact that the total population size of all species (**<https://www.codecogs.com/eqnedit.php?latex=N_T#0>**) is divided by the carrying capacity in the logistic term in equations 4 and 5. The shared carrying capacity between the species describes competition for general resources., and it should be better clarified when presenting the model. We have updated the description of the model in the main text to clarify this important point (Line 219):**

***“Note that in this formulation, the mutualistic partners share the same resources, as captured by the fact that the total population density of both species is included in the logistic term. This represents competition for general resources, such as available nutrients, space, etc.”***

Lines 257-259: Provide a more detailed description of the ‘two independent cooperating populations’. This was initially unclear to me.

**Thank you for the helpful comment. We updated the main text as follows (Line 279):**

***“In the absence of interspecies competition mutualisms have a decreased rescue probability that is similar to that of two independent cooperating populations - the rescue probability of two populations engaged in intraspecies cooperation but that do not interact with each other (since this is the rescue probability of two independent populations, it is given by the squared rescue probability of a single population).”***

There may be an issue in the jupyter notebook, Models.ipynb, as it does not work for me. I’m not sure if this is an issue on my end or if there is an error in the code. This is the error:

---------------------------------------------------------------------------

NameError Traceback (most recent call last)

in

----> 1 def simulate_cheaters(model=derivative,t=t,dt=dt,mu=mu,Nc=Nc,N1_initial=N1_initial,N2_initial=N2_initial,plot_bool=False,r1=r1,r2=r2,rm1=rm1,rm2=rm2,ro1=ro1,ro2=ro2,d_initial=d_initial,d_stress=d_stress,a=a,b=b,stress_onset=stress_onset,ax=None):

2 '''

3 Runs simulation of mutualism

4 model - The derivative

5 t - time of simulation

NameError: name 'ro1' is not defined

**Thank you for catching this error. We have mistakenly referenced a parameter in a code section where it was undefined. We have updated the code and corrected the issue.**
